# Mapping and validation of quantitative trait loci associated with concentrations of 16 elements in unmilled rice grain

**DOI:** 10.1007/s00122-013-2207-5

**Published:** 2013-11-15

**Authors:** Min Zhang, Shannon R. M. Pinson, Lee Tarpley, Xin-Yuan Huang, Brett Lahner, Elena Yakubova, Ivan Baxter, Mary Lou Guerinot, David E. Salt

**Affiliations:** 1 School of Biological Sciences, University of Aberdeen, Cruickshank Building, St Machar Drive, Aberdeen, Scotland AB24 3UU UK; 2 USDA-ARS, Dale Bumpers National Rice Research Center, 2890 Highway 130 East, Stuttgart, AR 72160 USA; 3 Department of Statistics, Purdue University, 150 N. University Street, West Lafayette, IN 47907-2067 USA; 4 Texas A&M AgriLife Research, Texas A&M University System, 1509 Aggie Dr., Beaumont, TX 77713 USA; 5 Department of Horticulture, Purdue University, 625 Agriculture Mall Dr., West Lafayette, IN 479072010 USA; 6 Horticulture and Landscape Architecture Department, Purdue University, West Lafayette, IN 47907 USA; 7 USDA-ARS Plant Genetics Research Unit, Donald Danforth Plant Science Center, St. Louis, MO 63132 USA; 8 Department of Biological Sciences, Dartmouth College, Hanover, NH 03755 USA

## Abstract

*****Key Message***:**

**QTLs controlling the concentrations elements in rice grain were identified in two mapping populations. The QTLs were clustered such that most genomic regions were associated with more than one element.**

**Abstract:**

In this study, quantitative trait loci (QTLs) affecting the concentrations of 16 elements in whole, unmilled rice (*Oryza sativa* L.) grain were identified. Two rice mapping populations, the ‘Lemont’ × ‘TeQing’ recombinant inbred lines (LT-RILs), and the TeQing-into-Lemont backcross introgression lines (TILs) were used. To increase opportunity to detect and characterize QTLs, the TILs were grown under two contrasting field conditions, flooded and irrigated-but-unflooded. Correlations between the individual elements and between each element with grain shape, plant height, and time of heading were also studied. Transgressive segregation was observed among the LT-RILs for all elements. The 134 QTLs identified as associated with the grain concentrations of individual elements were found clustered into 39 genomic regions, 34 of which were found associated with grain element concentration in more than one population and/or flooding treatment. More QTLs were found significant among flooded TILs (92) than among unflooded TILs (47) or among flooded LT-RILs (40). Twenty-seven of the 40 QTLs identified among the LT-RILs were associated with the same element among the TILs. At least one QTL per element was validated in two or more population/environments. Nearly all of the grain element loci were linked to QTLs affecting additional elements, supporting the concept of element networks within plants. Several of the grain element QTLs co-located with QTLs for grain shape, plant height, and days to heading; but did not always differ for grain elemental concentration as predicted by those traits alone. A number of interesting patterns were found, including a strong Mg–P–K complex.

**Electronic supplementary material:**

The online version of this article (doi:10.1007/s00122-013-2207-5) contains supplementary material, which is available to authorized users.

## Introduction

As one of the most important staple crops, rice provides more than 40 % of the daily calories for the world’s population (Parengam et al. 2010). Although rice is not considered a concentrated source of any particular mineral or vitamin, for those dependent on a rice subsistence diet, rice can be an important source of not just caloric energy, but also of vitamins and minerals important for human health. On the other hand, some elements, such as As and Cd, contained in grain and other food products can be harmful to human health if consumed in excess. Additional minerals, although not important for human health, impact the nutrition of the plant.

Despite the importance of essential minerals for human and plant health and despite the well-developed use of rice as a plant model for genetic analysis, the genetic mechanisms controlling the accumulation of the various mineral elements (*aka* the ionome) in rice remain largely unknown. Quantitative trait loci (QTLs) associated with seed ionomes have been reported in the genetic model species *Arabidopsis* *thaliana* (Baxter et al. 2012b; Bentsink et al. 2003; Waters and Grusak 2008), as well as in maize (Baxter et al. 2012a). Four rice studies have reported QTLs associated with accumulation of nutritional and potentially toxic elements in rice grain (Ishikawa et al. 2005; Lu et al. 2008; Norton et al. 2010; Stangoulis et al. 2007). The earliest of these rice studies (Ishikawa et al. 2005) utilized a set of chromosome segment substitution lines (CSSLs) and identified three QTLs associated with Cd concentration. Stangoulis et al. (2007) used liquid chromatography and inductively coupled plasma optical emission spectrometry to study the concentration of phytate and five elements (P, Fe, Zn, Cu and Mn) in a doubled haploid mapping population. Lu et al. (2008) used atomic absorption spectrometry to measure grain concentrations of Fe, Zn, Ca, Mn and Cu with which to identify grain element QTLs segregating within a set of rice recombinant inbred lines (RILs). Norton et al. (2010) used atomic absorption spectrometry to evaluate concentrations of Ca and Mg in rice grain and leaves, plus inductively coupled plasma mass spectrometry (ICP-MS) to evaluate an additional 15 elements to identify ionomic QTLs segregating among an F_6_ *indica* × *japonica* mapping population. The present study sought to identify QTLs affecting concentrations of one or more minerals in rice grain by studying two mapping populations derived from the same two parents with the anticipation of validating some of the QTLs through identification in both populations. Grain concentrations of 16 elements were observed simultaneously in order to test if QTL regions affected grain accumulation of individual or multiple elements. The first population was a set of ‘Lemont’ × ‘TeQing’ (*japonica* × *indica)* recombinant inbred lines (LT-RILs) similar in genetic structure to the mapping population analyzed by Norton et al. (2010), while the second population was a companion set of TeQing-into-Lemont backcross introgression lines (TILs) similar in genetic structure to the CSSLs observed by Ishikawa et al. (2005).

Flooding of rice paddies is known to alter soil chemistry and nutrient availability (Arao et al. 2009; Daum et al. 2002; Patrick and Khalid 1974; Patrick and Jugsujinda 1986; Zhang et al. 2004). Rice plant anatomy also differs depending on whether the plant is grown under flooded (anaerobic) or unflooded (aerobic) conditions (Hoshikawa 1989; Steffens et al. 2011; Uga et al. 2012). To enhance detection of QTLs affecting grain element concentrations, the TIL population was grown under two contrasting field conditions, flooded fields (reduced soil conditions) and regularly irrigated but unflooded fields (aerated soil conditions).

It is known that elements often accumulate more in one portion of the rice grain than others (Hansen et al. 2012; Liang et al. 2008; Lombi et al. 2009). Since the ratio of bran to endosperm (surface to volume) is greatly affected by grain shape (length, width, thickness), data on grain shape dimensions were included in the present study. Soil temperatures can impact soil chemistry and element availability, and air temperatures impact plant transpiration which can in turn affect nutrient uptake, transport, and accumulation in aerial portions of plants (Grusak and Pomper 1999; Quintana et al. 1999; Tani and Barrington 2005; Xiloyannis et al. 2001). Because temperatures are not constant throughout the Texas growing season, but are known to grow hotter from March through mid- to late-July, then cool again; date of flowering (a.k.a. heading date) was also recorded as a possible confounding factor due to different temperature regimes during grain-fill. In graminaceous plants, including rice, nutrients taken up by the roots are often not transported directly to the grain but are redirected to/from leaves or upward at the stem nodes which can result in a gradient of elemental concentration from lower to upper stems and leaves (Hoshikawa 1989; Kuramata et al. 2013; Uraguchi and Fujiwara 2012; Yamaji and Ma 2009). Data on plant height were included as well, because plant height is correlated with the number of culm nodes between roots and panicles (Samonte et al. 2005).

## Materials and methods

### Genetic materials

Putative ionomic QTLs were first identified in a set of 280 (RILs) derived from a cross between cultivars Lemont and TeQing hereafter referred to as the LT-RILs. Lemont (PI 475833) is a US tropical *japonica* rice cultivar with long-grain shape and earlier maturity than TeQing (PI 536047), an *indica* cultivar from China having medium-grain shape. The LT-RILs have been highly characterized and used in numerous QTL mapping studies including identification of loci affecting grain weight and shape (Li et al. 1997; Xu et al. 2004), grain yield (Li et al. 1997, 1998; Tong et al. 2006), plant architecture (Li et al. 1995, 1998, 1999b; Mei et al. 2005; Pinson et al. 2005), days to flowering (Pinson et al. 2005, 2012), and disease resistance (Li et al. 1999a; Pinson et al. 2005, 2010; Tabien et al. 2000, 2002). By mapping ionomic QTLs in this highly characterized population, the genomic location of newly identified ionomic loci could be readily compared with loci previously reported for other traits. Concentrations of LT-RIL grain elements were evaluated in seed samples produced and harvested over five different years, 2002, 2003, 2006, 2007 and 2008 at which time the field plants were in the F_15_, F_16_, F_17_, F_18_ and F_19_ generations, respectively.

The second mapping population in the study consisted of 123 TILs that contain molecularly tagged portions of the TeQing genome introgressed into the predominantly US tropical *japonica* genetic background of Lemont (Pinson et al. 2012). The TILs have been characterized for 159 simple sequence repeat (SSR) loci, and only backcross progeny found to contain ≥65 % Lemont alleles were included in the final set of 123 TILs. All portions of the TeQing genome are now represented across the set of TILs. Sets of chromosome segment substitution lines such as the TILs can also be used for de novo QTL mapping as well as QTL verification.

### Planting and phenotypic evaluation of the LT-RIL population grown under flooded field conditions

A total of five replications of LT-RIL seed were evaluated for grain element concentration, with one replication produced per year over 5 years (2002, 2003, 2006, 2007, and 2008). From five to seven repeated plots of the two parents, Lemont and TeQing, were randomized among the LT-RIL plots each annual replication. All LT-RIL plots were grown in Beaumont, TX under flooded paddy conditions, planted into soil classified as League clay (fine, smectitic, hypothermic Oxyaquic Dystrudert; USDA, 1999). Plots received 33.6 kg/ha P as preplant fertilizer and 73 kg/ha N (as urea) at the time of planting. Plots were drill-seeded approximately 2 cm deep using a Hege 80 Plot Seeder (Wintersteiger Ag, Dimmelstrasse 9, 4910 Ried/I., Austria). Germination was initiated when a flush irrigation moistened the soil. Additional flush irrigations were applied as needed to maintain soil moisture until seedlings were approximately 18 cm tall, at which time an 8–16 cm depth flood was applied and maintained on the fields until the last plots per field were harvested. Maintenance of the flood throughout the grain-fill periods of all genotypes was considered crucial since even short periods of field drainage have been shown to significantly impact soil chemistry, which in turn affected grain mineral concentrations, especially when the soil drainage occurred shortly before or during the grain-fill period (Arao et al. 2009; Daum et al. 2002).

The heading time of the earliest versus latest LT-RILs was previously found to differ by as much as 85 days (Pinson et al. 2005), causing concern that the early versus late heading LT-RILs would likely experience significantly different air and water temperatures during grain-fill, which would cause different plant transpiration and water uptake rates, and could potentially alter soil nutrient availability as well. In three of the five study years (2002, 2003, and 2006), the heading time between the various LT-RILs was better synchronized by dividing the population into four portions based on prior flowering observations, and planting them into neighboring field paddies at 7- to 10-day intervals with the RILs known to have longer intervals between seeding and flowering being planted first, and RILs known to flower in shorter time being planted last. This staggered planting resulted in more than 90 % of the LT-RILs flowering within a 3-week span in 2002, 2003, and 2006. Seeds of the LT-RILs and check varieties were drill-planted into plots consisting of 5 rows each, 2.4 m long, with 28 cm spacing between the rows within plots, as well as 28 cm spacing between the plots. Approximately 75 seeds were planted per row, with grain for analysis being obtained from the inner three rows of each 5-row plot. Approximately, 46,500 m^2^ of field space was required to plant each replication of the LT-RILs in 2002, 2003, and 2006.

In contrast, the field plots planted in 2007 and 2008 were planted in a manner that significantly decreased the field area (and presumably soil variance) within and between replications, but also necessitated that all plots be drill-seeded on a single day. The 2007 and 2008 field plots consisted of five seeds per genotype drill-seeded into 13-cm length lines, hereafter called hillplots. Five hillplots were planted per field-row with 60 cm between hillplots within each field-row, and 25 cm between rows. Each replication consisted of one plot per LT-RIL plus seven plots per parental genotype planted over a 67 m^2^ area in both 2007 and 2008.

The date of 50 % heading was recorded for each 5-row plot in 2002, 2003, and 2006, and per individual hillplot in 2007 and 2008. Days to heading (DHD) was calculated by subtracting the date of first irrigation after planting from the heading date recorded for each plot. All plots were hand harvested between 22 and 15 % grain moisture. Rough rice samples were dried to 12 % moisture using an ambient-forced-air drier and were then stored in sealed plastic boxes until phenotypic analysis.

### Planting and phenotypic evaluation of the TeQing-into-Lemont introgression lines (TILs) grown under both flooded and unflooded field conditions

The TILs were planted into hillplots in 2007 and 2008, and fertilized as described for the LT-RILs. Each TIL replication included 15 plots each of Lemont and TeQing. In order to study the putative ionomic QTLs under contrasting soil redox and plant development conditions, the TILs were grown in both flooded (anaerobic conditions) and unflooded (aerobic) fields. The unflooded fields were flush irrigated as needed to prevent water stress throughout the growing season, with water held on the field 5–14 h per flush. Two replications per water treatment were planted in both 2007 and 2008, but, due to storm damage, seed was harvested from two flooded plus one unflooded replications in 2007, and one flooded plus two unflooded replications in 2008. Heading date was observed and seed was hand harvested as previously described for the LT-RILs.

### Measurement of grain length, width and thickness

Rice grain dimensions (length, width, and thickness) of each of the LT-RILs and check varieties were measured using seed harvested in 2002 and 2003. TIL kernel dimensions were collected from seed harvested from a single flooded replication in each of 2 years, 2007 and 2008. For each plot, 100 whole brown rice kernels were produced with a Satake TH035A sheller (Satake Engineering Co. Ltd., Tokyo, Japan). They were then scanned with a WinSeedle Pro 2005aTM image analysis system (Regent Instruments Inc.; Sainte-Foy, Quebec, Canada) for determination of mean grain length (GL) and grain width (GW) in mm. Grain thickness (GT) of brown rice was measured in mm with a digital micrometer on a subset of 20 kernels. Since bran:endosperm ratio is strongly affected by kernel roundness, which is determined by a combination of these dimensions, we also approximated grain volume (GV) by multiplying GL × GW × GT. Kernel dimension data were averaged across the 2 years prior to QTL analysis.

### Analysis of grain element concentration

Using ICP-MS (Perkin-Elmer Elan DRCe ICP-MS), rice grain samples were simultaneously analyzed for concentrations of 16 elements. To validate the accuracy of the ICP-MS analytical method used in this study we first analyzed the NIST standard reference material 1568a (rice flour). The error reported on the NIST certified reference material for Mn, Fe, Cu, Zn, and Cd ranged from ±3 to ±12 %. The percentage of errors we observed, calculated as the NIST-certified value as compared to the value we obtained using our ICP-MS-based analysis for the same element, ranged from 5 to 13 %, and was not significantly different from the NIST expected errors. The convention in this report will be to report mineral concentrations as micrograms per gram grain dry weight (ppm), and to present the five plant macronutrients first, in high to lower order of their grain concentration, followed by the remaining 11 elements in alphabetical order. This results in the order of P, Mg, K, S, Ca, As, Cd, Co, Cu, Fe, Mn, Mo, Ni, Rb, Sr, Zn used in all tables and diagrams. Kernels for ICP-MS analysis were dehulled using a Satake TH035A sheller (Satake Engineering Co. Ltd., Tokyo, Japan) modified by replacing the rubber liner on the rollers with PU40 Polyurethane plastic. This modification prevented contamination of the rice samples with rubber particles, which are known to contain Zn (Stangoulis 2010).

We took two approaches to determine the optimal number of grains for ICP-MS analysis of each plot sample: a simulation approach based on single grain analysis and a purely theoretical simulation based approach. Because we were limited to parent data and to simplify the simulations, we focused on the power to detect difference between the two parents. It should be noted that this type of comparison is significantly less powerful than the QTL analysis performed with the RILs, but it allowed us to set a floor in terms of our detection power.

We started with a set of Lemont and TeQing seeds from a single 2006 planting date. Seed of these parental plots was hand harvested in two ways in 2006, collecting a set of five individual panicles as well as row-bulks (mix of all grain from ≥7 plants per row). The row-bulks were used for the preliminary studies, reserving the panicle harvests for the actual QTL study. After removing visibly green kernels, 10 grains were randomly selected from 6 and 3 row-bulks, respectively for Lemont and TeQing, and analyzed for ionomics as individual kernels (providing individual kernel data on 60 Lemont grains and 30 TeQing grains). The difference of the means of the two genotypes across all grains ranged from 0 to 77 % for the 16 elements. For each sample size between 3 and 20, we created 200 datasets by randomly picking samples from the parent data then used a *t* test to determine the significance level of the Lemont to TeQing difference for each dataset pairing (Supplemental Fig. 1). Analysis of the *n* = 3–20 subsets indicated that for some elements (e.g., Mg, Co, and Ni) even sample sizes as large as 20 were unlikely (<10 % of the time) to detect differences between Lemont and TeQing. In contrast, more than 95 % of the smallest subsamples (3 kernels) were able to detect a difference for Zn, followed by 60 % of the samples detecting a difference for As, Cu, and Mn. This indicated that samples as small as 3-kernels could support the identification of at least some grain element QTLs.

We also simulated datasets with three known levels of mean difference between each line (20, 50 and 100 %) and four levels of relative standard deviation (RSD) (10, 30, 50 and 100 %). These simulations showed that while a sample size of *n* = 3 would limit the power to detect small differences (10–20 %), especially for elements with high RSDs within the system, differences >50 % for elements with RSDs <30 % would be likely to be detected (Supplemental Fig. 2). Logistically, a three grain sample was the easiest size to handle (e.g., reliably digest completely) with the available equipment. As only two of the 16 elements (Ni and Cd) had RSDs >30 % and a 50 % difference could be easily fine-mapped in followup experiments, we chose to conduct all further analyses with three grain samples. Furthermore, the seeds phenotyped for the QTLs analyses were anticipated to be significantly more uniform per plot for grain maturity, size, and protein and starch chemistry than the row-bulks evaluated in the preliminary analyses due to careful sampling per plot as described below.

It has long been known that the rice grain starch and protein chemistry are impacted by the position of a rice grain along a single panicle (Dong et al. 2007; Liu et al. 2005) as well as differences in grain maturity and even air temperature during grain-fill (Harris and Juliano 1977; Resurreccion et al. 1977). Due to concern that that these factors might also be impacting grain element concentrations, the grains phenotyped for QTL analysis included only fully-mature kernels collected from the upper 1/4th (6 cm) of early-maturing panicles harvested per plot. Thus grains phenotyped for QTL analysis were expected to be from primary culms as opposed to later tillers, and significantly less variable per plot than the row-bulked seed used for the preliminary analyses. Approximately 20 kernels were selected from ≥3 panicles per plot harvest and dehulled. From this, three whole, non-diseased grains of mature brown rice were selected, weighed (approx. 0.05 g), and digested with 1.0-m1 concentrated HNO_3_ in 16 × 100 mm Pyrex tubes at temperatures stepped from ambient to 110 °C over a period of 12 h. Indium (EM Science) was added to the acid to a final concentration of 20 μg L^−1^ as an internal standard. Samples were diluted to 10.0 mL and analyzed on a PerkinElmer Elan DRCe ICP-MS (Perkin-Elmer Corp., Norwalk, CT, USA) for the following isotopes P31, K39, Mg25, S34, Ca43, As75, Cd114, Co59, Cu65, Fe57, Mn55, Mo98, Ni60, Rb85, Sr88, and Zn66. To normalize data between machine runs in order to correct for drift, portions of the samples were combined and used as a matrix-matched standard, measured after every nine samples. Samples were normalized to the averaged signals of the best-measured elements and weights of seven samples per run. All ICP-MS data on rice grain used in this study is publically available at http://www.ionomicshub.org.

### Analysis of variance (ANOVA)

One-way ANOVAs were conducted using JMP (version 9.0.0; SAS 2010) to partition the total phenotypic variance observed for each element into genetic, planting method, and year (nested within planting method) effects.

### Calculating trait means and correlations

Using SAS (version 9.1), the least squares (LS) means of each trait for each LT-RIL and parental genotype were calculated across the 5 years-replications, and across the three replications of TIL data, keeping the data from flooded and unflooded field conditions separate. Also, LS means were calculated for the LT-RILs grouped according to planting method, with the data from 2002, 2003, and 2006 representing staggered-planting into large plots, versus the 2007 and 2008 data representing hillplot planting on a single day per replication. Using JMP, Pearson’s correlation coefficients were calculated between each pair of all 16 elements, and between the 16 elements with grain shape, days to heading, and plant height. Heading date (DHD) and plant height (Ht, in cm) observed in a previous LT-RIL study (Pinson et al. 2005) were included in the LT-RIL correlations in addition to the DHD observed in the 2002–2006 stagger-planted plots. Likewise, TIL DHD data was from Pinson et al. (2012), and Ht data for the TILs came from yet another row-plot study (Pinson unpublished). Because the use of multiple comparisons can increase type I error, significance thresholds were determined using Bonferroni’s adjustments (Abdi 2007).

### Identification of QTLs in the Lemont–TeQing recombinant inbred population (LT-RILs)

Genotypic data consisted of 176 predominantly RFLP marker loci, determined in F_10_ LT-RILs by Tabien et al. (2000), and used previously in several disease resistance QTL mapping studies that utilized earlier generations of this LT-RIL population (Li et al. 1999a, b; Pinson et al. 2005, 2010; Tabien et al. 2000, 2002). The QTLs were analyzed using both multiple interval mapping (MIM) and Bayesian information criterion (BIC) analyses conducted on individual replication (year) data as well as on LS Means calculated per genotype across the multiple replications. QGene version 4.3.4 (Joehanes and Nelson 2008) was used to conduct single-trait MIM (Kao et al. 1999); BIC analyses were conducted as described by Zhang et al. (2005, 2008; Zhang 2010) using SAS 9.1.3 (SAS 2007). Because the results of the 5-year LS Mean MIM analyses provided comprehensive coverage of the QTLs identified in two or more years from MIM and BIC analysis of individual year data, and included all QTLs identified from analysis of LS means per planting method, only results from 5-year LS mean MIM analyses are included in the following tables and figures. The MIM analyses of trait LS means were conducted using a window size of 15 cM and a limit of three cofactors per element or single-trait MIM. The LOD thresholds for use in the MIM analyses were determined within QGene, using 1,000 permutations for each set of element data. The permuted LOD thresholds (*α* = 0.10) for all 16 elements proved quite similar across the years, ranging from 2.9 to 3.2. In that permuted LODs are estimates rather than definitive thresholds, it was decided to use a uniform LOD threshold of 3.0. The percentage of phenotypic variance explained by each QTL was equal to the generalized *R* ^2^ values calculated by the QGene single-trait MIM analyses (Nagelkerke 1991).

### Marker-trait associations among the TeQing-into-Lemont introgression lines (TILs)

Associations between the TIL SSR markers (markers determined by Pinson et al. 2012) and the observed grain traits were evaluated using the Marker-Trait Association module in JMP/Genomics 5.0 (SAS 2009). This module uses ANOVA to test for association between a trait and marker genotypes using a single marker at a time. The negative Log10 (NegLog10) conversion was used on all *p* values and the false discovery rate (FDR) multiple testing correction was calculated (Benjamini and Hochberg 1995; Weller et al. 1998) and applied to identify markers significantly associated (*α* = 0.1; *p* value threshold ≤0.0036) with one or more of the grain concentrations of the 16 elements. The markers with significant *p* values were then arranged according to their reported chromosomal locations (Pinson et al. 2012; Fig. 2). When multiple linked SSRs were found to have significant association with (and similar additive effect on) a grain element, the linkage association is indicated just once in the tables and figures, with the element names aligned by the marker that exhibited the highest association for that element in that genomic region beside dashed lines that indicate all markers found to be significantly associated with that and other linked elements. Each marker-trait ANOVA produced an estimated LS Mean value which is interpreted as the difference between the genetic effects of the TeQing/TeQing and the Lemont/Lemont (control) genotypes, which is also equal to 2× the additive effect. Physical locations of the SSR and RFLP markers reported in the Gramene database (http://www.gramene.org) were used to align the SSR and RFLP marker maps, and are reported here based on the markers’ Mbp locations on the Gramene Annotated Nipponbare Sequence 2009 map.

## Results

### Planting date affected DHD but not grain element concentrations among the stagger-planted LT-RILs

The staggered-planting dates employed in the 2002, 2003, and 2006 LT-RIL studies successfully narrowed the period of heading time and thus decreased the variance in day length, light intensity, and air and soil temperatures during grain-fill among the LT-RILs (results not shown). However, the potential for spatial variability in soil type and nutrient availability was considered high due to the staggered-planting dates across a large field area. Although differences due to planting date cannot be distinguished from locational soil differences between the staggered-planting blocks which were arranged from east to west each year, data from the 5 to 7 repeated plots of Lemont and TeQing that were included in each of the staggered-planting blocks were used to evaluate differences between the planting blocks per year. Within the repeated check plots observed in and across 2 years (2003 and 2006, no seed from 2002 check plots remained available for ionomic analysis), none of the 16 elements exhibited a consistent significant (*α* > 0.05) relationship with planting time or directional field location (data not shown). Consequently, concentrations of the 16 elements were not adjusted for within field location or planting time, but raw data used for further evaluation of means, variances, and correlations within, between and across years. In contrast, planting time was found to significantly and consistently affect the number of days from seeding to DHD. In all three stagger-planted years, the earlier planted Lemont and TeQing plots had longer vegetative stages than plots planted at later dates, likely due to a combination of longer day lengths and warmer temperatures as the season progressed. Lemont and TeQing similarly showed an average reduction in vegetative phase of 0.33 day for every day delay in planting across all 3 years. Thus, the Pearson correlations (Table 1) considered two sets of DHD data, one set observed from a prior study where the LT-RIL plots were planted on a single day (Pinson et al. 2005) in comparison with the DHD data collected from stagger-planted plots in 2002, 2003, and 2006.

**Table 1:**
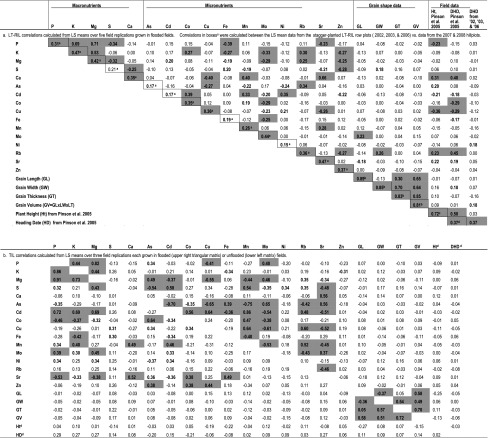
Correlations between traits in the LT-RIL and the TIL populations

Correlations between traits (grain element concentrations [ppm], grain shape, plant height, and days to heading) for the LT-RILs calculated from LS mean data across five field replications, all grown flooded. The averages of the pairwise correlations between the five LT-RIL years for each element are in the boxed cells. TIL data were analyzed separately for the flooded versus unflooded field conditions, and were grown only as hillplots in 2007 and 2008

Significance Levels: *Bold text* significant at the *α* = 0.05 level, *Shading of cells* indicates significance at the *α* = 0.01 level. Bonferoni adjustments were used to account for the multiple comparisons made

^a^Averages of the pairwise correlations between years reported in Supplementary Table 1

^b^The between-year correlations for grain shape attributes were calculated from measurements made on 2002-harvested seed versus shape data from seed harvested in 2003

^c^Correlations between the LT-RIL heading dates observed by Pinson et al. (2005) versus data presently collected from 2002, 2003, and 2006 stagger-planted plots

^d^TIL Height (Ht) and data on days to heading (DHD) data were from other field plots grown for other studies (Ht unpublished, DHD Pinson et al. 2012)

### Genotype and year contributed more to phenotypic variance than planting method

The proportion of phenotypic variance explained by genotype is also known as broadsense heritability (*H* ^2^). The average *H* ^2^among the 16 elements was 0.40, and was greater than 0.30 for all elements except Ni which had *H* ^2^ = 0.03 (Table 2). The highest heritabilities were seen for Mg, Mo and Sr which had *H* ^2^ > 0.50. On average, more variance was attributed to years than planting methods, but was again variable per element. Planting method explained <1 % of the variance observed for grain concentrations of Mg, Fe, and Rb. In contrast, for two of the 16 elements, As and Zn, planting method explained significantly more variance than years (1.5× more for As, 2.6× more for Zn). A different field location was planted each year, and because the stagger-planted 2002, 2003, and 2006 field plots were significantly closer to each other (maximum distance between plots across these years ≈250 m) than the field areas planted in 2007 and 2008 (≥900 m away), the proportions of variance attributed by ANOVA to ‘Planting method’ and ‘Year’ are both potentially confounded by differences in soil mineral availability. It is especially interesting, then, that that the pairwise correlations between years (Supplemental Table 1) shows that the year most closely correlated across all elements with either of the hillplot years (2007 and 2008) is 2002, a relatively distant stagger-planted year. The ANOVA and correlation results indicate that annual seasonal differences were a larger source of variance in grain element concentrations than were planting method or geographical distance. Accordingly, the remainder of the LT-RIL results and discussion focus on LS means calculated across all 5 years.

**Table 2:** Percentages of total phenotypic variance explained by genotype, planting method, and year

	Genotype^a^	Planting method^b^	Year^b^
P	34.3	3.4	28.6
K	32.5	18.9	27.1
Mg	52.4	0.2	11.7
S	30.4	13.8	12.2
Ca	46.9	1.1	14.3
As	25.4	18.9	12.6
Cd	30.1	7.8	7.2
Co	50.6	2.9	11.1
Cu	46.2	8.2	9.7
Fe	44.6	0.1	11.6
Mn	50.5	2.1	3.7
Mo	56.3	2.1	7.7
Ni	3.4	27.3	50.1
Rb	38.0	0.6	17.9
Sr	58.5	2.3	9.0
Zn	43.4	16.7	6.4
Avg. across elements	40.2	7.9	15.1

^a^The percentage of total phenotypic variance explained is also known as broadsense heritability (*H* ^2^)

^b^Because a different field location was used each year with the stagger-planted 2002, 2003, and 2006 fields being physically closer to each other than the field areas planted in 2007 and 2008, the variances attributed to ‘Planting method’ and ‘Year’ are both potentially confounded by differences in soil mineral availability. The ANOVA models considered ‘Year’ nested within ‘Planting method’

### Trait averages, ranges and correlations

**Fig. 1:**
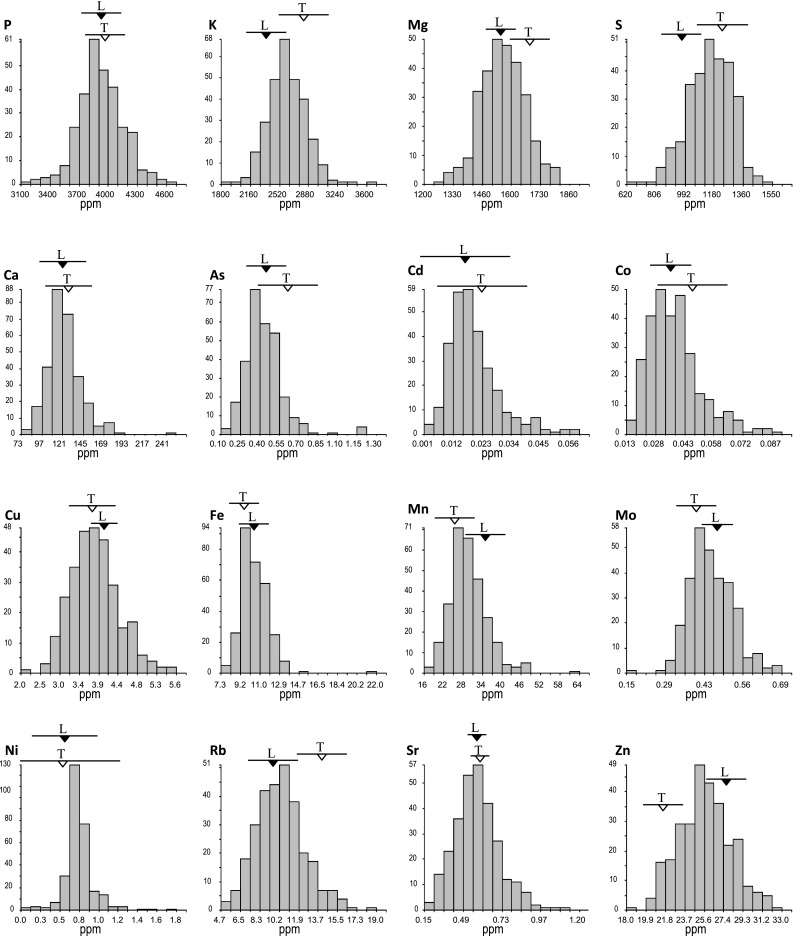
Histograms showing the range of the LS means observed among the population of 280 LT-RILs for the 16 elemental traits, all LT-RIL fields were flooded till all plots were mature and harvested. Elements are listed starting with the five macroelements (those reaching >100 ppm in rice grains), in order of their mean grain concentration, followed by the 11 remaining elements in alphabetical order. Also indicated are the LS Means (±1 standard deviation) of the multiple repeat check plots of the parental lines, Lemont and TeQing, grown in those same flooded fields

Histograms determined from the LS mean data calculated across all 5 years for each element concentration per LT-RIL (Fig. 1) show multiple LT-RILs with grain concentrations higher and/or lower than the parental means ± 1 standard deviation, indicating transgressive segregation among the LT-RILs for all 16 grain elements. For some elements (e.g. P, Cu, and Sr), transgressive segregation contributed significantly to the variance observed among the LT-RILs for that mineral, while for other elements (e.g. Ni and Fe) it was relatively minor. Transgressive segregation was observed among the LT-RILs even when the Lemont and TeQing parental values were not significantly different, e.g. P, Ca, Fe and Sr. These results suggested that the LT-RILs are segregating for genetic loci (QTLs) affecting the concentration of that element in rice grain, and that both Lemont and TeQing contributed alleles for increased element accumulation. Averages ± 1 standard deviations of the grain and plant traits observed in each individual year/replication among the parental lines, LT-RILs, and TILs can be viewed in Supplemental Table 2.

The Pearson correlations (*r*) determined between traits among the LT-RILs based on 5-year means are presented in Table 1a; correlations observed among the TILs (means across three replications) grown under flooded and unflooded field conditions are compared in Table 1b. The correlations shown in the boxes in Table 1a are the averages of the year-to-year correlations per element (Supplementary Table 1). Grain dimension traits exhibited the highest correlations between years. Among the elements, K, Mg, Mo, and Sr showed especially high correlation between years (*r* > 0.40), while As, Cd, Fe, and Ni exhibited more sensitivity to environmental variance. While not a perfect correlation, these elements were also generally among those determined by ANOVA to have the most extreme heritabilities (Supplementary Table 1).

Some interesting patterns can be seen among the correlations between the various elements. Correlation between three essential macronutrients P–K, P–Mg, and Mg–K are highly significant and positively correlated in both the LT-RILs and the TILs, under both flooded and unflooded conditions (Table 1). Other correlations that were significant over all populations, planting method, and field water treatments include Ca–Sr (positive), Fe–K (negative), and Mn–Cd (positive). Of the 42 element–element correlations that were significant among the LT-RILs, 21 (50 %) proved significant also among the TILs. The dataset revealing the highest number of significant element–element correlations was that of the TIL population grown under flooded field conditions.

Among the TILs, whether grown flooded or unflooded, none of the grain elements were significantly correlated with grain shape, plant height, or DHD. In contrast, among the LT-RILs, Mo and Sr were significantly correlated with grain length, while S and Rb were significantly correlated with grain width. The DHD as observed in the stagger-planted LT-RIL plots was significantly correlated with just one element, Ni, while DHD as measured from LT-RIL plots planted on a single day (Pinson et al. 2005) correlated with seven elements, namely, Ca, Cd, Co, Cu, Fe, Rb, and Sr. Plant height and heading are known to be correlated among the LT-RILs (Pinson et al. 2005), and five of the elements correlated with both DHD and height.

### QTLs affecting grain element concentration identified among LT-RILs and TILs

**Fig. 2:**
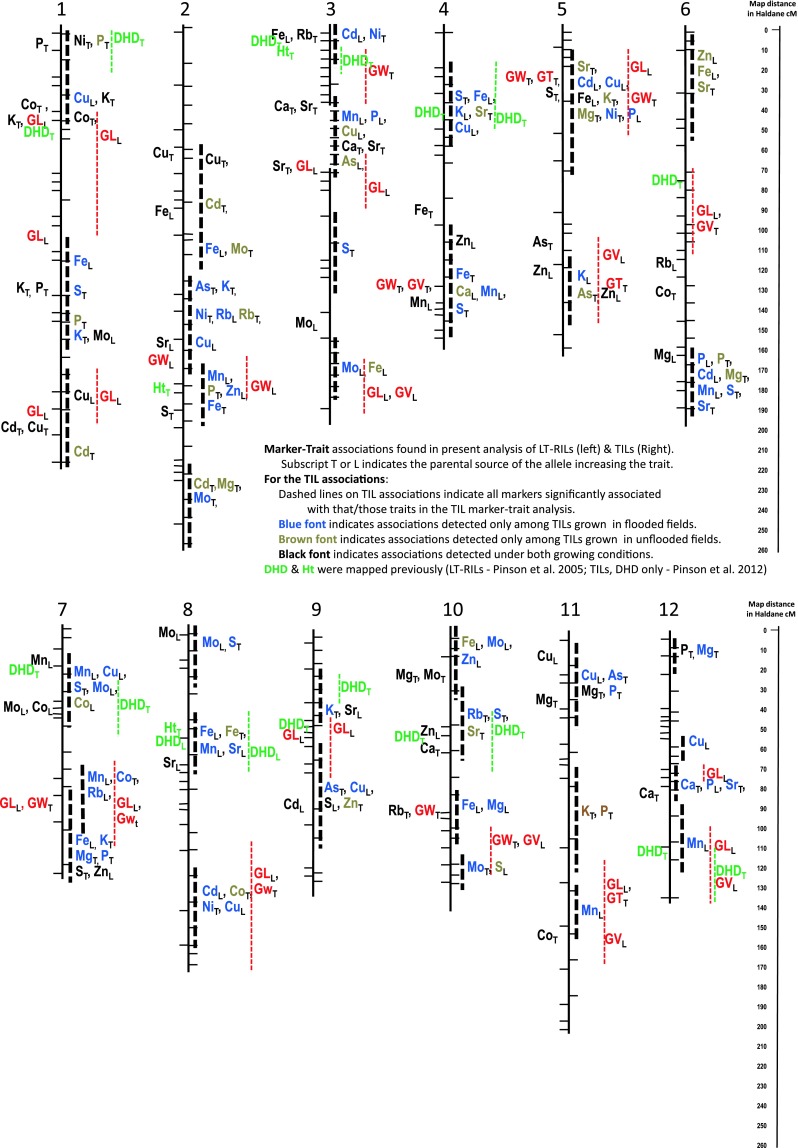
A total of 127 QTLs for grain element content were identified among the LT-RIls and the TILs. QTLs found significant among the LT-RILs are indicated to the *left* of the chromosome lines; QTLs identified among TILs are to the *right*. QTLs for grain element concentration often clustered in chromosomal regions as diagrammed here, and as indicated in Table 3. QTLs identified for grain shape dimensions are also indicated, as are QTLs for plant height and days to heading, which were determined in prior studies (Pinson et al. 2005, 2012)

Between the LT-RIL and the TIL analyses, a total of 134 putative QTLs for concentration of specific elements in rice grain were identified (Table 3; Fig. 2). The LT-RIL MIM LOD peaks are indicated by element names to the left of the chromosomes in Fig. 2. Results of the TIL marker-trait analyses are to the right of the chromosomes in Fig. 2, with the element names placed beside the marker that exhibited the strongest statistical association and color coded to indicate significance among flooded TILs (blue), unflooded TILs (brown), or both (black). Mapping populations consisting of introgression lines (ILs) such as the presently utilized TILs and the CSSLs in which Ishikawa et al. (2005) identified Cd QTLs, that consist of progeny lines having a common (shared) genetic background into which one or few chromosomal segments have been substituted with chromosome pieces (genes) from another source, allow scientists to observe precisely the phentoytpic effect of one or few genes per trait per line. This allows QTLs of smaller individual effect to be detected as statistically significant, and accordingly, our QTL analyses detected more QTLs segregating among TILs than among LT-RILs. However, because IL mapping populations generally contain less recombination within each chromosomal region than one finds in a RIL mapping population, the QTLs are not mapped with as much precision. The QTL location estimates determined here among the TILs are not considered precise, as indicated by the dashed lines in Fig. 2.

**Table 3:** QTLs showing main effects in the LT-RIL and/or TIL analyses

Elements having signif. assoc.			Chr.	Approx. cM	Approx. Mbp	LT-RIL detected linkages				TIL detected linkages					All elements plus DHD & Ht loci ^c,d^ mapped to this region among LT-RILs and/or TILs	QTLs affecting grain element concentration identified elsewhere in other populations
										Flooded			Unflooded			
LT-RILs	TILs-Flooded	TILs-Unfl.				LOD^a^	Add. effect^b^; − = L, + = T	%Var (from *R* ^2^)	RFLP Marker closest to LT-RIL LOD peak	SSR locus most strongly associated among TILs	Prob. of linkage^a^	Add. effect^b^; − = L, + = T	Prob. of linkage^a^	Add. Effect^b^; − = L, + = T		
Macronutrients in order of concentration (ppm) in rice grains																
P		P	1	0–8	0–3	3.3	53.7	4.7	RG472	RM495			0.0035	115	Ni, P, DHD^d^	
P		P	1	110–122	23–35	3.6	55.6	5.2	CDO118	RM3411			0.0036	106	Fe, K, Mo, P, S, GL	Cu^f,^ Zn^g^
		P	2	160–194	24–31					RM106			0.0036	144	Cu, Fe, Mn, P, S, Sr, Zn, GW, Ht^c^	Cd^e^, Mo^f^
	P		3	gap, 28–70	5–17					RM156	0.0012	−70.0			As, Ca, Cu, Mn, P, Sr, GL,	Cd^f,i^, Mg^f^, Mo^f^
	P		5	0–20	0–5.5					RM289	0.0034	−104.1			Cd, Cu, K, Mg, Ni, P, S, Sr, GL, GW, GT	Zn^e^
	P	P	6	148–173	24–31					RM340/OSR 21	0.0030	−62.9	0.0032	95.6	Cd, Mn, Mg, P, S, Sr	P^f^
	P		7	70–102	26–30					RM248	0.0032	46.8			Fe, K, Mg, P, S, Zn	K^f^, P*i* ^g^
	P		11	0–25	0–5					RM26073	0.0036	87.6			As, Cu, Mg, P	Mn^f^
		P	11	63–100	15–19					RM229			0.0028	116	K, P	
	P		12	0–15	0–2					RM5568	0.0008	115.1			Mg, P	Zn^e^, Pb^f^
	P		12	65–95	16–22					RM3448	0.0005	−109.2			Ca, Cu, P, Sr, GL, Ht^c^	Cu^f^
K	K	K	1	34–40	3–7	9.4	88.2	12.9	RG532a	RM1	0.0036	59.2	0.0030	88.3	Co, Cu, K, GL, DHD^c^	
K	K		1	110–122	23–35	4.1	67.7	5.8	CDO455	RM5501	0.0036	51.7			Fe, K, Mo, P, S, GL	Cu^f,^ Zn^g^
	K		2	130–170	17–25					RM341	0.0023	63.1			As, Cu, K, Ni, Rb, Sr, GW	Cu^e^
	K		4	15–52	5–20					RM3317	0.0028	−54.8			Cu, K, Fe, S, Sr, DHD^c,d^	Ca^e^
		K	5	0–20	0–5.5					RM13			0.0010	162	Cd, Cu, K, Mg, Ni, P, S, Sr, GL, GW, GT	Zn^e^
	K		5	98–138	21–27					RM188	0.0022	−72.1			As, K, Zn, GT, GV	Ca^e^, P^g^, IP6 ^g^
	K		7	70–102	26–30					RM248	0.0036	39.5			Fe, K, Mg, P, S, Zn	K^f^, P*i* ^g^
	K		9	12–38	7–15					RM3912	0.0036	50.5			K, Sr, GL, DHD^c,d^	Fe^e^, P^f^, Mo^f^, Se^f^
		K	11	63–100	15–19					RM229			0.0032	79.4	K, P	
		Mg	2	210–240	33–38					RM166			0.0036	48.0	Cd, Mg, Mo	lpa^h^
		Mg	5	0–20	0–5.5					RM13			0.0016	66.5	Cd, Cu, K, Mg, Ni, P, S, Sr, GL, GW, GT	Zn^e^
Mg		Mg	6	148–152	24–31	3.4	−22.7	4.9	RZ682	OSR 21			0.0032	35.8	Cd, Mn, Mg, P, S, Sr	P^f^
	Mg		7	70–102	26–30					RM248	0.0007	25.0			Fe, K, Mg, P, S, Zn	K^f^, P*i* ^g^
Mg			10	0–36	0–12	3.0	−25.4	4.1	G1084	RM467					Fe, Mg, Mo, Zn	Cd^f^, Mn^f^
	Mg		10	65–88	18–21					RM5494	0.0034	−33.0			Fe, Mg, Rb, GW	
Mg	Mg		11	15–33	0–5	7.1	37.1	9.9	RZ781	RM332	0.0020	36.4			As, Cu, Mg, P	Mn^f^
	Mg		12	0–15	0–2					RM5568	0.0027	42.4			Mg, P	Zn^e^, Pb^f^
	S		1	98–145	23–35					RM246	0.0029	29.0			Fe, K, Mo, P, S, GL	Cu^f,^ Zn^g^
S			2	186–194	26–31	3.4	38.4	4.9	RZ273	RM266					Cu, Fe, Mn, P, S, Sr, Zn, GW, Ht^c^	Cd^f^, Mo^f^
	S		3	103–111	25–29					RM55	0.0018	26.7				Co^f^, Se^f^
	S		4	15–52	5–20					RM307	0.0023	25.1			Cu, K, Fe, S, Sr, DHD^c,d^	Ca^e^
	S		4	87–132	28–33					RM317	0.0013	27.2			Ca, Fe, Mn, S, Zn, GV	Fe^f^, P^f^, Pb^f^
S			5	0–20	0–5.5	3.2	39.0	4.6	gl	RM13					Cd, Cu, K, Mg, Ni, P, S, Sr, GL, GW, GT	Zn^e^
	S		6	148–173	24–31					OSR 21	0.0014	25.9			Cd, Mn, Mg, P, S, Sr	P^f^
	S		7	10–30	5–20					RM214	<.0001	32.4			Co, Cu, Mn, Mo, S, DHD^c,d^	Cd^f^, Fe^f^, Zn^f^
	S	S	7	70–102	26–30					RM248	0.0021	21.1	0.0014	45.3	Fe, K, Mg, P, S, Zn	K^f^, P*i* ^g^
	S		8	0–10	0–2					RM408	<.0001	38.4			Mo, S	
	S	S	9	90–120	16–22					RM215	0.0031	−24.8	0.0031	−48.9	As, Cd, Cu, S, Zn	Ca^e^, Cd^f^, Mg^f^
	S		10	34–44	15–18					RM25553–2	0.0029	34.6			Ca, Rb, S, Sr, Zn, DHD^c,d^	Zn^f^
		S	10	88–103	21–23					RM1374			0.0034	−66.3	Mo, S, GW, GV	Zn^f^
Ca	Ca	Ca	3	22–28	4–6	4.7	5.0	6.7	RG100	RM5626/RM16	0.0031	7.0	0.0034	4.8	As, Ca, Cu, Mn, P, Sr, GL	Cd^f,i^, Mg^f^, Mo^f^
		Ca	4	87–132	28–33					RM348			0.0012	−6.9	Ca, Fe, Mn, S, Zn, GV	Fe^f^, P^f^, Pb^f^
Ca			10	34–44	15–18	3.1	3.6	3.1	CDO98	RM25470					Ca, Rb, S, Sr, Zn, DHD^c,d^	Zn^f^
Ca	Ca		12	65–95	16–22	3.1	4.8	3.1	G402	RM3448	<.0001	13.5			Ca, Cu, P, Sr, GL, Ht^c^	Cu^f^
Micronutrients in alphabetical order, units ppm																
	As		2	130–170	17–25					RM341	0.0028	0.058			As, Cu, K, Ni, Rb, Sr, GW	Cu^e^
		As	3	gap, 28–70	5–17					RM16			0.0036	−0.007	As, Ca, Cu, Mn, P, Sr, GL	Cd^f,i^, Mg^f^, Mo^f^
As		As	5	80–98	21–27	3.0	0.034	3.7	RG13	RM421			0.0034	0.009	As, K, Zn, GT, GV	Ca^e^, P^g^, IP6^g^
	As		9	90–120	16–22					RM1189	0.0005	0.061			As, Cd, Cu, S, Zn	Ca^e^, Cd^f^, Mg^f^
	As		11	0–25	0–5					RM167	0.0012	0.063			As, Cu, Mg, P	Mn^f^
Cd		Cd	1	176–184	40–44	4.4	0.002	6.3	RG236	RM6840			0.0031	0.013	Cd, Cu, GL	Fe^e^, Cd^f^, Fe^f^, P^f^, Pb^f^, Se^f^, IP5 ^g^
		Cd	2	61–85	5–9					RM6840			0.0031	0.013	Cd, Cu, Fe, Mo	Cu^f^
		Cd	2	210–240	33–38					RM166			0.0020	0.014	Cd, Mg, Mo	lpa (low phytic acid)^h^
	Cd		3	0–8	0–3					RM132	0.0029	−0.004			Cd, Fe, Ni, Rb, GW, DHD^c,d,^ Ht^c^	MIPS^h^
	Cd		5	0–20	0–5.5					RM13	0.0005	−0.004			Cd, Cu, K, Mg, Ni, P, S, Sr, GL, GW, GT	Zn^e^
	Cd		6	148–173	24–31					OSR 21	0.0034	−0.002			Cd, Mn, Mg, P, S, Sr	P^f^
	Cd		8	102–144	23–28					RM149	0.0030	−0.003			Cd, Co, Cu, Ni, GL, GW	
Cd			9	90–120	16–22	4.0	−0.002	5.8	RG570	RM215					As, Cd, Cu, S, Zn	Ca^e^, Cd^f^, Mg^f^
Co	Co	Co	1	26–36	3–7	7.1	0.004	10.0	RG140	RM490	0.0012	0.005	0.0002	0.007	Co, Cu, K, GL, DHD^c^	Mn^e,g^, Se^f^, Mo^f^
Co			6	116–128	17–22	3.6	0.003	5.2	RG424						Co, Rb	Cd^i^
Co		Co	7	22–26	5–20	3.8	−0.003	5.5	RG678	RM2			0.0031	−0.004	Co, Cu, Mn, Mo, S, DHD^c,d^	Cd^f^, Fe^f^, Zn^f^
	Co		7	59–77	25–27					RM118	0.0011	0.003			Co, Mn, Rb, GL, GW	K^f^, Zn^e^, P*i* ^g^
		Co	8	102–144	23–28					RM447			0.0036	0.005	Cd, Co, Cu, Ni, GL, GW	
Co			11	120–150	19–22	3.1	0.003	3.5	RZ537b	RM206					Co, Mn, GL, GV	Mg^f^
	Cu		1	0–40	0–7					RM1	0.0004	−0.21			Co, Cu, K, GL, DHD^c^	
Cu	Cu	Cu	1	178–184	40–44	3.0	0.10	3.2	RG236	RM3482	0.0025	−0.21	0.0017	−0.53	Cd, Cu, GL	Fe^e^, Cd^f^, Fe^f^, P^f^, Pb^f^, Se^f^, IP5 ^g^
Cu	Cu	Cu	2	54–62	5–9	8.4	0.21	11.7	RG83	RM6378	0.0035	0.14	0.000	0.66	Cd, Cu, Fe	Cu^f^
	Cu		2	130–170	17–25					RM13556	0.0006	−0.22			As, Cu, K, Ni, Rb, Sr, GW	Cu^e^
		Cu	3	gap, 28–70	5–17					RM156			0.0005	−0.34	As, Ca, Cu, Mn, P, Sr, GL	Cd^f,i^, Mg^f^, Mo^f^
	Cu		4	15–52	5–20					RM1359	0.0035	−0.21			Cu, K, Fe, S, Sr, DHD^c,d^	Ca^e^
	Cu		5	0–20	0–5.5					RM13	0.0035	−0.22			Cd, Cu, K, Mg, Ni, P, S, Sr, GL, GW, GT	Zn^e^
	Cu		7	10–30	5–20					RM214	0.0019	−0.16			Co, Cu, Mn, Mo, S, DHD^c,d^	Cd^f^, Fe^f^, Zn^f^
	Cu		8	102–144	23–28					RM3155	0.0006	−0.18			Cd, Co, Cu, Ni, GL, GW	
	Cu		9	90–120	16–22					RM1189	0.0023	−0.16			As, Cd, Cu, S, Zn	Ca^e^, Cd^f^, Mg^f^
Cu	Cu		11	0–25	0–5	4.0	−0.16	5.7	RG1022	RM167	<.0001	−0.29			As, Cu, Mg, P	Mn^f^
	Cu		12	46–61	7–11					RM27982–2	0.0035	−0.15			Ca, Cu, P, Sr, GL, Ht^c^	Cu^f^
	Fe		1	98–145	23–35					RM5	0.0035	−0.29			Fe, K, Mo, P, S, GL	Cu^f,^ Zn^g^
Fe	Fe		2	80–99	7–10	3.6	−0.31	5.2	RG437	RM452	0.0022	−0.24			Cd, Fe, Mo	Fe^g^
	Fe		2	160–194	24–31					RM6933	0.0036	0.32			Cu, Fe, Mn, P, S, Sr, Zn, GW, Ht^c^	Cd^f^, Mo^f^
Fe			3	0–4	0–3	3.0	−0.25	3.9	RG104	RM132					Cd, Fe, Ni, Rb, GW, DHD^c,d^, Ht^c^	MIPS^h^
		Fe	3	134–174	29–36					RM514			0.0036	−1.2	Fe, Mo, GL, GV	Fe^f^
	Fe		4	15–52	5–20					RM3317	0.0035	−0.25			Cu, K, Fe, S, Sr, DHD^c,d^	Ca^e^
Fe	Fe		4	78–88	24–25	3.1	0.26	3.3	RZ740b	RM3217	0.0034	0.24				Mg^f^
		Fe	5	0–20	0–5.5					RM13			0.0036	−2.3	Cd, Cu, K, Mg, Ni, P, S, Sr, GL, GW, GT	Zn^e^
		Fe	6	0–41	0–4					RM190			0.0036	−2.2	Cd, Fe, Sr, Zn	Pb^f^, Se^f^, Zn^f^
	Fe		7	70–102	26–30					RM248	0.0036	−0.21			Fe, K, Mg, P, S, Zn	K^f^, P*i* ^g^
	Fe	Fe	8	35–52	5–12					RM44/RM1148	0.0031	−0.30	0.0035	1.4	Fe, Mn, Sr, DHD^c,d^, Ht^c^	Fe^g^
		Fe	10	0–36	0–12					RM222			0.0028	−1.1	Fe, Mg, Mo, Zn	Cd^f^, Mn^f^
	Fe		10	65–88	18–21					RM1108	0.0035	−0.37			Fe, Mg, Rb, GW	
	Mn		2	160–194	24–31					RM106	<.0001	−2.31			Cu, Fe, Mn, P, S, Sr, Zn, GW, Ht^c^	Cd^f^, Mo^f^
	Mn		3	22–28	4–6					RM517	0.0035	−2.13			As, Ca, Cu, Mn, P, Sr, GL	Cd^f,i^, Mg^f^, Mo^f^
Mn	Mn		4	126–134	28–33	3.1	−1.26	4.5	RZ590b	RM348	0.0031	−0.71			Ca, Fe, Mn, S, Zn, GV	Fe^f^, P^f^, Pb^f^
	Mn		6	148–173	24–31					OSR21	0.0035	−1.61			Cd, Mn, Mg, P, S, Sr	P^f^
Mn	Mn		7	0–8	10–14	8.0	−2.01	11.2	RG30	RM214	0.0017	−1.42			Co, Cu, Mn, Mo, S, DHD^c,d^	Cd^f^, Fe^f^, Zn^f^
	Mn		7	59–77	25–27					RM234	0.0003	−1.30			Co, Mn, Rb, GL, GW	K^f^, Zn^e^, P*i* ^g^
	Mn		8	35–52	5–12					RM22559	0.0002	−2.88			Fe, Mn, Sr, DHD^c,d^, Ht^c^	Fe^g^
	Mn		11	120–150	19–22					RM21	0.0029	−1.25			Co, Mn, GL, GV	Mg^f^
	Mn		12	70–110	23–27					RM3739	0.0036	−1.40			Mn, GL, GV, DHD^c,d^	Fe^g^, Zn^g^
	Mo	Mo	1	98–145	23–35					RM5501	0.0017	−0.026	0.0035	−0.021	Fe, K, Mo, P, S, GL	Cu^f,^ Zn^g^
		Mo	2	85–110	7–10					RM452			0.0036	0.025	Cd, Fe, Mo	Fe^g^
	Mo		2	210–240	33–38					RM166	0.0035	0.027			Cd, Mg, Mo	lpa^h^
Mo	Mo		3	134–174	29–36	3.7	−0.020	5.3	RZ761	RM422	0.0015	−0.029			Fe, Mo, GL, GV	Fe^f^
Mo	Mo		7	22–26	5–20	3.9	−0.017	5.6	G20	RM11	0.0031	−0.021			Co, Cu, Mn, Mo, S, DHD^c,d^	Cd^f^, Fe^f^, Zn^f^
Mo	Mo		8	0–10	0–2	3.7	−0.017	5.4	C424b	RM408	<.0001	−0.037			Mo, S	Cd^i^
Mo	Mo		10	0–36	0–12	3.0	0.022	4.1	G1084	RM3311	0.0030	−0.022			Fe, Mg, Mo, Zn	Cd^f^, Mn^f^
	Mo		10	88–103	21–23					RM5494	0.0035	0.037			Mo, S, GW, GV	Zn^f^
	Ni	Ni	1	0–40	0–7					RM495	0.0036	0.11	0.0036	0.21	Ni, P	
	Ni		2	130–170	17–25					RM475	0.0026	0.17			As, Cu, K, Ni, Rb, Sr, GW	Cu^e^
	Ni		3	0–8	0–3					RM132	0.0036	0.18			Cd, Fe, Ni, Rb, GW, DHD^c,d^, Ht^c^	MIPS^h^
	NI		5	0–20	0–5.5					RM13	0.0004	0.20			Cd, Cu, K, Mg, Ni, P, S, Sr, GL, GW, GT	Zn^e^
	Ni		8	102–144	23–28					RM149	0.0035	0.12			Cd, Co, Cu, Ni, GL, GW	
	Rb	Rb	2	130–170	17–25					RM475	0.0036	−0.65	0.0035	1.3	As, Cu, K, Ni, Rb, Sr, GW	Cu^e^
Rb			3	2–8	0–3	8.9	0.79	12.3	C515	RM489					Cd, Fe, Ni, Rb, GW, DHD^c,d^, Ht^c^	MIPS^h^
Rb			6	109–115	10–19	3.0	−0.47	4.2	G1468b						Co, Rb	Cd^i^
	Rb		7	59–77	25–27					RM118	0.0014	−0.44			Co, Mn, Rb, GL, GW	K^f^, Zn^e^, P*i* ^g^
	Rb		10	34–44	15–18					RM25470	0.0030	0.66			Ca, Rb, S, Sr, Zn, DHD^c,d^	Zn^f^
Rb			10	65–88	18–21	3.1	0.63	4.5	CDO98	RM147					Fe, Mg, Rb, GW	
Sr			2	156–168	21–25	5.2	−0.036	7.4	G45	RM3688					As, Cu, K, Ni, Rb, Sr, GW	Cu^e^
Sr^i^	Sr^j^	Sr	3	22–28	4–6	3.5	0.030	5.1	RG100^i^	RM156^j^	<.0001	0.045	0.0015	0.026	As, Ca, Cu, Mn, P, Sr, GL	Cd^f,i^, Mg^f^, Mo^f^
Sr^i^			3	70–76	17–24	6.0	0.039	8.4	RG482^i^							
		Sr	4	15–52	5–20					RM3317			0.0034	0.030	Cu, K, Fe, S, Sr, DHD^c,d^	Ca^e^
		Sr	5	0–20	0–5.5					RM1024			0.0016	0.064	Cd, Cu, K, Mg, Ni, P, S, Sr, GL, GW, GT	Zn^e^
		Sr	6	0–41	0–4					RM225			0.0035	0.039	Cd, Fe, Sr, Zn	Pb^f^, Se^f^, Zn^f^
	Sr		6	148–173	24–31					OSR 21	0.0011	0.042			Cd, Mn, Mg, P, S, Sr	P^f^
Sr	Sr		8	58–64	7–14	3.0	−0.025	3.8	G1314a	RM44	0.0022	−0.032			Fe, Mn, Sr, DHD^c,d^, Ht^c^	Fe^g^
	Sr	Sr	9	12–38	7–15					RM3912	0.0034	−0.031	0.0031	−0.030	K, Sr, GL, DHD^c,d^	Fe^e^, P^f^, Mo^f^, Se^f^
		Sr	10	34–44	15–18					RM25553–2			0.0028	0.042	Ca, Rb, S, Sr, Zn, DHD^c,d^	Zn^f^
	Sr		12	65–95	16–22					RM3448	<.0001	0.069			Ca, Cu, P, Sr, GL, Ht^c^	Cu^f^
	Zn		2	160–194	24–31					RM106	0.0036	−0.82			Cu, Fe, Mn, P, S, Sr, Zn, GW, Ht^c^	Cd^f^, Mo^f^
	Zn	Zn	4	87–132	28–33					RM317	0.0002	−0.98	0.0012	−1.4	Ca, Fe, Mn, S, Zn, GV	Fe^f^, P^f^, Pb^f^
Zn	Zn	Zn	5	102–126	21–27	5.7	−0.73	8.1	CDSR49	RM421	0.0031	−0.48	0.0031	−0.79	As, K, Zn, GT, GV	Ca^e^, P^g^, IP6 ^g^
		Zn	6	0–41	0–4					RM435			0.0035	−1.5	Cd, Fe, Sr, Zn	Pb^f^, Se^f^, Zn^f^
	Zn	Zn	7	70–102	26–30					RM248	<.0001	−0.95	0.0035	−1.0	Fe, K, Mg, P, S, Zn	K^f^, P*i* ^g^
		Zn	9	90–120	16–22					RM3909			0.0021	1.1	As, Cd, Cu, S, Zn	Ca^e^, Cd^f^, Mg^f^
	Zn		10	0–38	0–12					RM222	0.0032	−0.68			Fe, Mg, Mo, Zn	Cd^f^, Mn^f^
Zn	These RIL & TIL QTLs overlap		10	34–44	15–18	3.0	−0.54	4.4	RG241a	RM147					Ca, Rb, S, Sr, Zn, DHD^c,d^	Zn^f^
Grain length, width and thickness measured in mm, volume was approximated by multiplying GL × GW × GT																
GL	GL	n/a	1	34–42	6–23	3.1	−0.15	4.1	RG532a	RM8208	<.0001	−0.31	n/a		Co, Cu, K, GL, DHD^c^	
GL	GL	n/a	1	82–90	23–35	3.1	−0.13	3.3	CDO348	RM156	0.0001	−0.21	n/a		Fe, GL	
GL	GL	n/a	1	172–184	40–44	4.4	−0.19	6.3	RZ801	RM104	0.0006	−0.31	n/a		Cd, Cu, GL	Cd^f^, Fe^e,f^, P^f^, Pb^f^, Se^f^, IP5 ^g^
GL	GL	n/a	3	74–80	17–24	4.6	−0.18	6.5	RG482	RM156	0.0001	−0.21	n/a		As, Ca, Cu, Mn, P, Sr, GL	Cd^f,i^, Mg^f^, Mo^f^
	GL	n/a	3	144–174	32–37					RM85	<.0001	−0.26	n/a		Fe, Mo, GL, GV	Fe^f^
	GL	n/a	5	24–38	1–7					RM13	0.0022	−0.28	n/a		Cd, Cu, K, Mg, Ni, P, S, Sr, GL, GW, GT	Zn^e^
	GL	n/a	6	67–104	4–10					RM19691	<.0001	−0.33	n/a		GL, GV, DHD^c^	
GL	GL	n/a	7	59–77	25–27	3.1	−0.13	3.3	CDO497	RM478	<.0001	−0.28	n/a		Co, Mn, Rb, GL, GW	K^f^, Zn^e^, P*i* ^g^
	GL	n/a	8	102–144	23–28					RM149	0.0002	−0.25	n/a		Cd, Co, Cu, Ni, GL, GW	
GL	GL	n/a	9	12–38	11–17	3.0	0.11	2.7	CDO395	RM409	0.0036	−0.19	n/a		K, Sr, GL, DHD^c,d^	Fe^e^, P^f^, Mo^f^, Se^f^
	GL	n/a	11	120–150	19–22					RM21	<.0001	−0.26	n/a		Co, Mn, GL, GV	Mg^f^
	GL	n/a	12	65–95	16–22					RM277	0.0026	−0.21	n/a		Ca, Cu, P, Sr, GL, Ht^c^	Cu^f^
	GL	n/a	12	70–122	23–27					RM3739	0.0005	−0.23	n/a		Mn, GL, GV, DHD^c,d^	Fe^g^, Zn^g^
GT		n/a	5	24–38	1–7	5.2	0.032	7.4	gl	RM13			n/a		Cd, Cu, K, Mg, Ni, P, S, Sr, GL, GW, GT	Zn^e^
	GT	n/a	5	85–120	20–27					RM161	0.0036	−3.77	n/a		As, K, Zn, GT, GV	Ca^e^, P^g^, IP6 ^g^
	GT	n/a	11	120–150	19–22					RM21	0.0019	0.018	n/a		Co, Mn, GL, GV	Mg^f^
GW	GW	n/a	2	160–174	23–30	3.0	−0.050	4.2	C624x	RM13556	0.0004	−1.41	n/a		As, Cu, K, Ni, Rb, Sr, GW	Cu^e^
	GW	n/a	3	0–8	0–3					RM489	0.0015	0.076	n/a		Cd, Fe, Ni, Rb, GW, DHD^c,d^, Ht^c^	MIPS^h^
GW		n/a	4	116–122	28–33	3.0	−0.036	3.0	RG214	RM317			n/a		Ca, Fe, Mn, S, Zn, GV	Fe^f^, P^f^, Pb^f^
GW	GW	n/a	5	24–38	1–7	8.7	0.078	12.0	Y1049	RM437	<.0001	0.093	n/a		Cd, Cu, K, Mg, Ni, P, S, Sr, GL, GW, GT	Zn^e^
GW	GW	n/a	7	59–77	25–27	8.2	0.079	11.4	CDO497	RM478	<.0001	0.052	n/a		Co, Mn, Rb, GL, GW	Zn^e^, P*i* ^g^
	GW	n/a	8	102–144	23–28					RM149	0.0005	0.061	n/a		Cd, Co, Cu, Ni, GL, GW	
GW	GW	n/a	10	88–103	21–23	3.2	0.044	4.7	RG752	RM228	0.0019	0.059	n/a		Mo, S, GW, GV	Zn^f^
	GV	n/a	3	144–174	32–37					RM85	<.0001	−1.20	n/a		Fe, Mo, GL, GV	Fe^f^
GV		n/a	4	116–122	28–33	3.0	−1.02	4.2	RG214	RM348			n/a		Ca, Fe, Mn, S, Zn, GV	Fe^f^, P^f^, Pb^f^
	GV	n/a	5	85–120	20–27					RM421	<.0001	0.027	n/a		As, K, Zn, GT, GV	Ca^e^, P^g^, IP6 ^g^
	GV	n/a	6	67–104	4–10					RM19691	0.00029	−1.23	n/a		GL, GV, DHD^c^	
	GV	n/a	10	88–103	21–23					RM228	0.0032	−3.77	n/a		Mo, S, GW, GV	Zn^f^
	GV	n/a	11	120–150	19–22					RM206	0.0028	–3.86	n/a		Co, Mn, GL, GV	Mg^f^
	GV	n/a	12	70–122	23–27					RM17	0.0036	−3.82	n/a		Mn, GL, GV, DHD^c,d^	Fe^g^, Zn^g^

QTLs for multiple elements tended to cluster, with each genomic region being associated with more than one element, as shown in the column 2nd from the right. Similarly mapped grain element QTLs reported elsewhere (Ishikawa et al. 2005; Lu et al. 2008; Norton et al. 2010; Stangoulis et al. 2007) are also indicted in the right-most column

^a^The QTL significance thresholds were as follows: permutated LOD thresholds for the RIL QTLs were 3.0 for *α* = 0.1, and 5.1 for for *α* = 0.05. For the TILs, the *α* = 0.1 *p* value threshold was 0.0036

^b^Units for additive effects are ppm for all elements; days for days to heading (DHD); mm for grain length (GL), width (GW), and thickness (GT); and mm^3^ for grain volume (GV)

^c^Mapping of days to heading (DHD) and plant height (Ht) among the LT-RILs was by Pinson et al. 2005

^d^DHD QTLs were mapped among the TILs by Pinson et al. 2012, data was collected only from flooded field plots

^e^Similarly mapped grain element QTL mapped by Lu et al. (2008)

^f^Similarly mapped grain element QTL mapped by Norton et al. (2010)

^g^Similarly mapped grain element QTL mapped by Stangoulis et al. 2007. IP5 stands for inositol-5-phosphate, IP6 for inositol-6-phosphate, P*i* for inorganic P (H_2_PO_4_)

^h^Location of a low phytic acid locus (*lpa*) and a locus affecting L-myo-inositol 1-phosphate synthase (MIPS) reported by Larson et al. 2000

^i^Similarly mapped grain element QTL mapped by Ishikawa et al. (2005), study focused on identification of Cd QTLs among a set of chromosome segment substitution lines

^j^While the LT-RIL analysis indicated the possibility of two Sr QTLs on each end of a marker gap, the improved molecular tagging of this region among the TILs indicated instead a single Sr QTL

Analysis of the LT-RILs, flooded TILs, and unflooded TILs each identified 40, 92, and 47 specific grain element QTLs, respectively. Interestingly, the flooded TILs, where the most ionomic QTLs were identified, is also the population wherein the most significant element-to-element correlations were determined (Table 1). Non-genetic variance was higher among the unflooded replications than the flooded replications, with the coefficients of variance for the 16 elements among the multiple Lemont and TeQing check-plots planted per replication being, on average, twofold higher in the unflooded replications than the flooded replications (data not shown). One impact of such an increase in non-genetic variance would be reduced ability to identify QTLs of smaller phenotypic effect, consistent with the identification of fewer QTLs among the unflooded TILs than among flooded TILs. This was most dramatically demonstrated by Mn for which no QTLs were identified among unflooded TILs.

Confidence in a particular QTL increases when it is validated with identification in more than one population or study-environment. In this study, QTLs could be considered validated by identification among both LT-RILs and among the TILs for the same element. Alternatively, identification of a particular QTL among both the flooded and unflooded TILs would not only validate that QTL, but would also reflect the stability of that QTL across widely divergent field environments. The three left-hand columns in Table 3 indicate the populations and environments in which each QTL was found statistically significant. Among the 134 QTLs, six were considered strongly validated and stable in that they were found significantly associated with the same element among the TILs grown both flooded and unflooded as well as in the LT-RILs. For example, a QTL for K was identified on chromosome 1 in the LT-RILs and in the TILs grown under both flooded and unflooded field conditions (all three population/environment combinations). The estimated additive effects (88, 60 and 88) were fairly consistent across the populations and field environments, and this stable and validated QTL explained 13 % of the phenotypic variance observed among the 5-year LS means of the LT-RILs. This QTL also achieved a significant LOD in all five of the individual year MIM analyses. The stability of this QTL across populations and environments is also indicated in Fig. 2 where it is shown that the TeQing allele was associated with increased grain-K concentration in the LT-RILs, and in the TILs grown under both flooded and unflooded conditions. The TeQing allele of the closely linked Co QTL was also associated with increased grain-Co in all three population/environment conditions. In contrast, the nearby QTL for which the Lemont allele significantly increased grain-Cu among the flooded TILs was not validated in a second population or environment (Fig. 2; Table 3), nor was it validated with identification of a similarly located Cu QTL reported by others (Table 3).

Additional QTLs that were validated with stable expression across all study populations and environments include a QTL for Cu located in the 5–7 Mbp (54–62 cM) region of chromosome 2 that explained 12 % of the LT-RIL variance for Cu and was similarly located with a Cu-QTL reported previously by Norton et al. (2010), a QTL affecting both Ca and Sr in the 4–20 Mbp region of chromosome 3 that explained 5–8 % of the LT-RIL variance for these two elements, and a Zn QTL in the lower 21–27 Mbp region of chromosome 5 that explained 8 % of the LT-RIL variance for Zn. The sixth QTL identified in all three populations/environments was a Cu QTL in the 40–44 Mbp region of chromosome 1, but curiously, the TeQing allele was found associated with increased grain-Cu among the LT-RILs while the Lemont allele was found associated with increased Cu among both the flooded and unflooded TILs.

Thirty-three additional QTLs were validated by identification in two of the three study populations/environments. For example, the Mg QTL at the top (0–5 Mbp) of chromosome 11 was found significant among flooded LT-RILs and TILs, but was not significant among unflooded TILs. While this QTL acquired a significant LOD in only two of the two of the five individual LT-RIL years (2002 and 2007), it exhibited a sub-threshold LOD peak in additional years such that the MIM analyses based on LS means calculated across individual planting methods also identified this QTL as statistically significant (LOD > 3.0) in both the stagger-planted and hillplot analyses. While the inability to identify this QTL as significant among unflooded TILs might have been due to increased non-genetic variance among unflooded plots, it could also be related to differences in soil chemistry, root architecture, and/or plant physiology between flooded and unflooded field conditions. At least one QTL per each of the 16 elements was validated in two or more populations/environments. This is true even for Ni, for which no QTLs were identified among the LT-RILs. Among the elements, Mo appeared most stable among years and populations, with 71 % (5 of 7) of its QTLs being found significant in at least two populations/environments.

The phenotypic data used to identify QTLs among the LT-RILs was collected over five field environments, one replication per environment. While high rates of environmental variance within this data would prevent identification of some QTLs, those identified in this manner would be expected to be highly stable. Indeed, a majority (27/40 = 67 %) of the QTLs identified among the LT-RILs based on across-replication LD Means were validated among the TILs. Furthermore, for six elements (As, Cu, Mn, Mo, Sr and Zn) each and every QTL identified among the LT-RILs was identified also among the TILs. It cannot be explained, then, why some QTLs with high significance in one population were not found significant in the other. For example, the Rb QTL identified among the LT-RILs on chromosome 3 with a highly significant 5-year LOD of 8.9, was significant in four of the five individual year LT-RIL MIM analyses, but was not even close to statistically significant among the TILs.

As a corollary to the fact that the flooded TILs identified the most QTLs overall, a lower portion (31/92 = 34 %) of the QTLs identified among the flooded TILs were also found significant among the LT-RILs (also flooded). Of the 121 specific element QTLs identified among the TILs (either flooded or unflooded or both), 26 (21 %) were verified due to co-location with a QTL for the same element found significant among the LT-RILs, and an additional 10 (8 %) were verified with significance in both the flooded and unflooded TILs, leaving 86 (71 %) unverified in the present study. However, when element QTLs reported in the literature are considered (rightmost column of Table 3), another 7 QTLs are verified with identification in two or more populations/environments. For example, the P QTL region on chromosome 7 overlapped with a P QTL reported previously by Stangoulis et al. (2007). Likewise, external but not internal verification exists for one K QTL (chromosome 7, Norton et al. 2010), one Cd QTL (chromosome 9, Norton et al. 2010), two Cu QTLs (chromosomes 2 and 12, Lu et al. 2008 and Norton et al. 2010, respectively), one Fe QTL (chromosome 3, Norton et al. 2010), and one Zn QTL (chromosome 6, Norton et al. 2010). Overall, 36 % (43) of the QTLs found significant among the TILs were verified in terms of association with the same element in more than one population or environment.

One concern associated with interpretation of ICP-MS ionomics data is that interference from molecular ionic species generated in the ICP-MS plasma can decrease the accuracy of some element measurements, such as Ca. In contrast, Sr is measured more reliably by ICP-MS and is known to be a chemical analog to Ca, sharing plant uptake and transport mechanisms and even serving as acceptable substitute in some biochemical processes. Therefore, determination of Sr, a chemical analog of Ca, in the seed samples could prove to be a more reliable indicator of Ca-uptake and transport capabilities than direct observation of Ca. Among the LT-RILs, a Ca QTL mapped to the end of an RFLP gap on chromosome 3, while Sr mapped to both ends of this marker gap, suggesting the possibility of a large Sr QTL actually residing inside the RFLP gap. This is indeed supported by the results of the flooded and unflooded TILs, which indicated that both Ca and Sr were most strongly associated with the region between RM5626 and RM16, near the lower end of the LT-RIL RFLP gap. Among both the LT-RILs and the TILs, the TeQing allele at this locus increased Sr by 0.04, and increased Ca by 7 ppm (Table 3). If the concept of QTL verification is extended to include co-location of QTLs for different but chemically analogous elements, then the Ca QTL on chromosome 10 can be considered validated by the similarly located Sr QTL, raising the number of validated Ca QTLs from 2 to 3 (out of 4 total). Similarly, the co-located P and As QTLs on chromosomes 3 and 11 along with the co-located K and Rb QTLs on chromosome 2 would also thus be considered confirmed.

### QTL regions commonly associated with more than one element

The 134 individual element QTLs were often found linked (<25 cM) or grouped into multi-element clusters as seen in Fig. 2, and noted in Table 3. Clustering of QTLs for multiple elements was especially common among the TILs. For example, LT-RIL analysis detected a single QTL for S in a region of chromosome 5 where TIL analysis detected association with eight elements, though S was not among them (Table 3; Fig. 2). Using a linkage threshold of 25 cM, the 134 QTLs in Fig. 2 merge into approximately 39 clusters or genomic regions (four on chromosome 1, five on chromosome 2, four on chromosome 3 and so on). Further study would be required to determine if co-location of QTLs is due to existence of linked but separate genes, shared regulatory genes, or pleiotropy (one gene affecting multiple phenotypes). However, considering recent reports of element networks within plants (Baxter 2009; Baxter et al. 2008, 2012b), it seems likely that the QTL clusters are sometimes due to a single factor affecting multiple elements. For example, rates of Se uptake have been shown to affect uptake of Co and Cu in both pean and wheat plants (Landberg and Greger 1994). This lends support to the present identification both a Co and Cu QTL in the 0–7 Mbp region of chromosome 1 (Table 3, Fig. 2) where a rice Se QTL was previously reported to reside (Norton et al. 2010).

### Co-location of QTLs among the highly correlated macronutrients, Mg, P, and K

Mg, P and K do not share uptake mechanisms, are not tightly chemically coupled with respect to soil solution levels in flooded vs. unflooded soil conditions (De Datta 1987), and do not appear connected in a common plant network among vegetative tissues (Baxter et al. 2008; Baxter 2009), yet they were found highly correlated among the grains of LT-RILs and the TILs, whether grown flooded or unflooded. Furthermore, of the 11 QTLs for P (Table 3; Fig. 2), five were also associated with Mg concentration among the LT-RILs and TILs, and another P locus, on chromosome 3, was co-located with a Mg locus reported by Norton et al. (2010). Of the 11 QTLs for P, 4 were co-located with K loci, and another two were linked with K loci (i.e., different LOD peaks but near each other on the same chromosome). There were two loci associated with all three elements, P, Mg and K, one each on chromosomes 5 and 7. These were also the only two loci with a common association with Mg and K. The nature of these QTLs, however, does not always fit nicely with their expected nature based upon the positive correlations observed among these three elements across the three studies. For example, for the QTL region on chromosome 5, the allele that increased P decreased Mg and K. Interestingly, the gene action for the P and Mg QTL located near RM340 and OSR21 on chromosome 6 was opposite between the flooded and unflooded TILs, with the Lemont allele being associated with an increase in both P and Mg under flooded conditions, but with the TeQing allele being associated with higher P and Mg under unflooded conditions.

### Co-location of grain element QTLs with grain shape QTLs, but gene action often indicated opposite allelic effects

A sphere has a lower surface to volume ratio than an elongated ovoid shape. Likewise, a rice kernel having a rounder short- or medium-grain shape has a lower bran:endosperm ratio than a long-grained rice. Since the various elements accumulate in different concentrations in different portions of the grain (Hansen et al. 2012; Liang et al. 2008; Lombi et al. 2009) it was hypothesized that some of the observed differences in concentration of specific elements in brown (whole) grain might be attributed to differences in grain shape as indicated by GL, GW, GT, and estimated GV. Upon first observation, there does appear to be a correlation between QTLs for grain shape and element concentration with all 20 grain shape loci being located near QTLs for grain element content (Fig. 2; Table 3). Furthermore, chromosomal regions associated with grain shape were more often associated with high numbers of elements. Of the 11 regions found to be associated with five or more elements in the present study, eight also contained QTLs affecting grain length, width, and/or thickness.

However, upon deeper investigation, the gene actions of co-located grain shape and element QTLs were often opposite from that expected if the elemental concentration differences were due solely to a change in grain shape. For example, K is generally contained in higher concentrations in the outer bran layer than in the rice endosperm (Lombi et al. 2009), suggesting that alleles associated with increased grain length would be associated also with increased grain-K concentration. However, for the region of chromosome 1 near RG532a and RM1 that was associated with GL, K, Cu, and Co, the Lemont allele increased grain length but decreased grain-K concentration. Likewise, the chromosomal region further down on chromosome 1 shows correlation with GL, Fe, K, P, and S. Bran is generally considered more concentrated for Fe, K, and P than endosperm (Liang et al. 2008; Lombi et al. 2009). The Lemont allele in this region was associated with increased Fe as one would expect from an increased grain length and bran:endosperm ratio, but conversely associated with decreased K and P. Upon examinations such as these for all regions associated with grain shape and element concentration, it appears that the majority, if not all, of the grain element QTLs detected within the present study cannot be explained solely by association with grain shape, and are thus detecting other mechanisms of element uptake, transport, and/or grain accumulation.

### Co-location with heading time previously mapped within the LT-RILs and TILs

Because plants with a longer vegetative phase (later heading dates) have a longer window of opportunity to mine nutrients out of the soil, it raises the question of whether or not late heading is associated with increased grain element concentration. Alternatively, later grain-fill periods generally occur under cooler air and soil/water temperatures, which could also impact root uptake rates, and rates of translocation of elements within plants. As shown in Table 3 and Fig. 2, nine QTLs have been reported for DHD among the LT-RILs (Pinson et al. 2005), and eight among the TILs (Pinson et al. 2012). The two prior DHD studies had seven DHD loci in common, resulting in a total of 10 DHD loci, two on chromosome 1 which are near each other but were mapped to different locations by the LT-RILs and TILs, plus one DHD QTL each on chromosomes 3, 4, 6, 7, 8, 9, 10, and 12. Only one DHD QTL, on chromosome 6, was not associated with any elemental QTLs. In looking for relationships or patterns of correlation between DHD and the 16 elements, we find primarily a lack of consistency or relationship. For example, the DHD QTL at the top of chromosome 1 was associated with P, but since only one of 12 P QTLs was associated also with DHD, there does not appear to be a causative relationship between DHD and P. The element having QTLs most often co-located with DHD QTLs was Sr, with four (out of 10) QTLs linked with DHD QTLs. An increase in Sr was associated with increased DHD in three cases, in one case greater Sr concentration was associated with decreased DHD, and there were six additional Sr loci not associated with DHD. The two elements having Pearson correlations with DHD >0.40 among the LT-RILs (Table 1a) were Ca and Rb. The detection of correlation between DHD and Rb likely traces to the fact that the Rb QTL having the largest individual effect (explained 12 % of total LT-RIL variation for Rb, Table 3) is located on the top of chromosome 3 where one of the DHD QTLs with largest effect is known to reside (Pinson et al. 2005, 2012). Since Ht also maps to this region, this Rb QTL probably also underlies the weak correlation (*r* = 0.23, Table 1a) noted among the LT-RILs between Ht and Rb.

### Differences in element accumulation observed under flooded versus unflooded conditions

Soil chemistry and nutrient availability are known to differ between aerobic (oxidized) and anaerobic (chemically reduced) soil conditions, with some elements being more available to plant uptake when they are chemically reduced under anaerobic soil conditions, e.g. Fe and As. Consistent with soil chemistry and nutrient availability predictions, concentrations of As in grains of TILs and parental plots grown under flooded conditions were 10-times higher than seen in rice produced under unflooded conditions (Supplemental Table 1). Contrary to what one would expect based on increased availability of Fe in soil solution under flooded conditions, grain concentrations were not significantly different between the flooded and unflooded plots (supplemental table 1). Another factor altering nutrient uptake is the fact that rice roots, when grown under flooded conditions, diffuse oxygen which can reoxidize elements in the rhizosphere. Reoxidation of iron in the soil solution causes a coating of ferric hydroxide to form on the rice roots known as an iron plaque. The iron plaques that form on several wetland plant species in addition to rice have been shown to affect the uptake of several nutrients, increasing the uptake of some elements (e.g. P, Zhang et al. 1999), but forming a barrier that can decrease plant uptake of others (e.g. Zn, Zhang et al. 1998; Se, Zhou et al. 2007; As, Hu et al. 2005). Furthermore, rice root architecture also differs when plants are grown under flooded as opposed to unflooded conditions, with plants producing more surface-level roots under flooded conditions but deeper roots under unflooded growth conditions (Hoshikawa 1989; Uga et al. 2012).

We analyzed seeds of the TIL population grown in both flooded and unflooded hillplots in 2007 and 2008 in order to investigate whether the grain-element QTLs identified under one field condition have similar importance and allelic effects under the contrasting water treatment. In four instances, the parental allele that was associated with increased element uptake under flooded conditions was conversely associated with decreased concentration of that element in grains produced by unflooded TILs: 1) at a QTL for Rb located on chromosome 2, 2) at a QTL for Fe concentration located on chromosome 8, 3) at a QTL for P concentration located on the bottom of chromosome 6, which is co-located with 4) a QTL for Mg on chromosome 6 wherein the Lemont allele was associated with increased Mg among the LT-RILs, grown under flooded conditions, while the TeQing allele was associated with increased Mg among the TILs grown unflooded, but was not found significantly associated among the flood-produced TILs (Table 3; Fig. 2). These instances of altered gene action observed under flooded and unflooded growing conditions, while rare among the presently identified grain element QTLs, do suggest the importance of controlling soil saturation levels when conducting ionomic studies.

## Discussion

The correlations noted between the grain elemental concentrations are of interest because they suggest the possibility of shared mechanisms/genes for root uptake and/or transport to rice grains. For example, although Sr is not essential to plants, it is taken up by plants because it is a chemical analog of Ca. Therefore, the fact that Sr and Ca were highly correlated under both flooded and unflooded conditions (*r* ranged 0.52–0.66, Table 1) was as anticipated. In contrast, Mg, P, and K each have different mechanisms of root uptake and transport within plants, and yet were so highly correlated (*r* ranged 0.44–0.91) with each other across both populations and water-management treatments (Table 3), that their correlations with the other 13 elements also tended to be similar in both magnitude and sign (positive or negative). Elements that were typically negatively (or not significantly) correlated with Mg, P, and K among the LT-RILs and TILs, under both flooded and unflooded growing conditions were Ca, Sr, Fe, Cu and Zn, while Rb and Cd were predominantly positively correlated. The relationship between some elements reversed sign depending on whether the seed was produced under flooded or unflooded conditions. Specifically, S and Ni were negatively (or not significantly) correlated with the Mg–P–K complex among both the LT-RILs and the TILs when grown under flooded conditions but were positively correlated when grown unflooded, while As and Co were positively correlated with Mg–P–K under flooded conditions, but negatively correlated in seed from unflooded field conditions.

Strong correlations among P, Mg and K have also been reported in seeds of *A. thaliana* (Baxter et al. 2008; Vreugdenhil et al. 2004) and maize (Baxter et al. 2012a). In rice, as in many cereal grains (Bryant et al. 2005), most of the seed phosphorus is found in the form of a mixed K/Mg salt of phytic acid in the germ and aleurone layer, so one possible explanation of the positive P–Mg–K correlations might be that they are all driven by phytate levels in the grain. Vreugdenhil et al. (2004) found co-localization of QTL for K and Ca with a high P/high phytate locus in *Arabidopsis* suggesting that phytate levels were affecting P–K associations. However, a phytate-less mutant of rice did not display altered total grain P concentration (Bryant et al. 2005), suggesting that something other than phytate levels in the bran is controlling total grain accumulation of P.

From the present study, 134 QTLs associated with the concentration of individual elements in rice grains were found clustered in 39 chromosomal regions, suggesting the importance of considering grain ionomes and element networks rather than focusing future studies on one or few targeted elements. The interconnectedness observed between the elements also indicates the importance of conducting ionomics studies under conditions that minimize within-study variance for factors affecting soil fertility such as soil moisture, soil texture, content of organic matter, and soil temperature. Even small irregularities or dips in soil surface level may cause moisture and nutrients to pool, causing micro-environmental variance in nutrient availability between two nearby plants.

More QTLs achieved statistical significance in the analysis of flooded TILs than in the analysis of LT-RILs or unflooded TILs. This may be, in part, due to the fact that the more homogenous genetic background among the TILs enhances ability to detect QTLs of smaller individual effect. Additionally, three of the five LT-RIL replications involved plots grown over a wide field area, likely subjecting them to increased within-study variance for soil fertility. An increase in environmentally induced variance for grain element concentration would result in decreased ability to detect grain element QTLs of smaller effect among the LT-RILs. Field flooding increases spread of soil nutrients from one point in the field to another [with the relatively increased hydraulic conductivity of the saturated soil (Hillel 1980)], decreasing micro-environmental effects. This may be why more QTLs were detected among the flooded TILs than among the unflooded TILs.

Grain shape, heading time, and plant height could all potentially induce differences in element concentrations observed among whole (unmilled) grains. Many of the elemental QTLs, even when co-located with QTLs for grain shape, HD, or Ht, did not differ as predicted if the grain elemental concentration differences were caused solely by these other traits. This suggests that those grain element QTLs have an underlying cause that is different from, and stronger than, the predicted shape, HD, or Ht effects.

Mapping the grain element QTLs in the LT-RILs and TILs made it possible to readily compare the location of the newly identified grain element QTLs with those of several agronomic traits. Most of the previous LT-RIL and TIL gene-mapping efforts targeted the identification of loci associated with disease resistance. The locations were thus known for several loci conferring resistance to rice sheath blight disease (Pinson et al. 2005), rice blast disease (Tabien et al. 2000, 2002), bacterial leaf blight (Li et al. 1999a), and bacterial panicle blight (Pinson et al. 2010). Interestingly, the disease resistance loci themselves were often clustered or co-located (Pinson et al. 2010). When the locations of the previously mapped disease resistance QTLs were compared to the newly identified grain ionomic QTLs, an association between Ca, Sr and disease resistance was indicated. Of the 11 QTLs associated with Ca and/or its chemical analog, Sr, 10 had mapped to genomic regions known to contain loci affecting resistance to one or multiple rice diseases. The sole Sr locus not associated with disease resistance was located on the upper end of chromosome 6, and was detected among unflooded TILs but not among the flooded LT-RILs and TILs, leaving this QTL region unverified. It is known that Ca increases strength of cell walls and membranes (for review of Ca in plants, see Hepler 2005), and thus may contribute to quantitative disease resistance by slowing infection rates. It is also known that Ca plays a role in the cell-to-cell signaling critical to the hypersensitive necrosis that provides resistance to rice blast disease and rice bacterial blight disease (Kuta and Gaivaronskaya 2004).

In summary, QTLs are here reported for concentrations of 16 elements (ionome) in rice grain. Two mapping populations, the LT-RIL and the TIL (which was grown both flooded and unflooded), were used in the study so that putative QTLs could be identified in one, and verified in the other. This constitutes the first report of grain ionomic QTLs identified in seed produced under unflooded field conditions. Transgressive segregation was observed in the LT-RIL population for all 16 elements. A number of interesting patterns were found among the correlations between elements deserving of additional study to understand their bases. These include a Ca and Sr association that was largely supported by the QTL associations and a strong Mg–P–K complex. The QTLs also showed interesting patterns deserving additional study. For example, the elemental QTLs tended to cluster into genomic regions (134 QTLs into 39 regions) often with multiple elements associated with a region, lending support to the concept of ionomic networks. This further suggests that, when studying grain nutritional value, it is important to study multiple elements at a time, and to carefully control factors such as soil fertility, temperature, and pH that can affect uptake the ability of plants to take nutrients in from the soil. More QTLs and element-to-element correlations were identified among the flooded TILs than the unflooded TILs, hinting that less microenvironmental variability might exist under flooded conditions. Several plant characteristics, namely grain shape, heading time and plant height, had much less direct influence on rice grain mineral concentrations based on the QTL associations than was anticipated. In addition, a strong association of Ca/Sr QTLs with disease resistance loci was noted.

## Electronic supplementary material

Below is the link to the electronic supplementary material.

Supplementary material 1 (PPT 636 kb)

Supplementary material 2 (PPT 287 kb)

Supplementary material 3 (DOC 278 kb)

Supplementary material 4 (DOC 67 kb)

## Abbreviations

BIC: Bayesian information criterion
cM: Centimorgans
CIM: Composite interval mapping
CSSL: Chromosome segment substitution line
DHD: Days from planting to heading (also known as flowering)
ICP-MS: Inductively coupled plasma mass spectrometry
LOD: Logarithm of the odds ratio
*lpa*: Low phytic acid
LS Mean: Least squares mean
Mbp: Mega base pairs, indicates physical location on a chromosome
LT-RIL: Recombinant inbred line derived from ‘Lemont’ × ‘TeQing’
MIM: Multiple interval mapping
MIPS: *Myo*inositol 1-phosphate synthase
ppm: Parts per million
QTL: Quantitative trait locus (QTLs, quantitative trait loci)
RFLP: Restriction fragment length polymorphism
RIL: Recombinant inbred line
SSR: Simple sequence repeat
TIL: A TeQing-into-Lemont backcross introgression line
