# Supplementary material for: Mapping and validation of quantitative trait loci associated with concentrations of 16 elements in unmilled rice grain
Source: Theor Appl Genet. 2013 Nov 15;127(1):137–65. doi: 10.1007/s00122-013-2207-5 (PMC4544570; doi:10.1007/s00122-013-2207-5)
Supplement: Supplementary file 1 — Supplementary material 1 (PPT 636 kb) [file 122_2013_2207_MOESM1_ESM.ppt]

## Slide 1
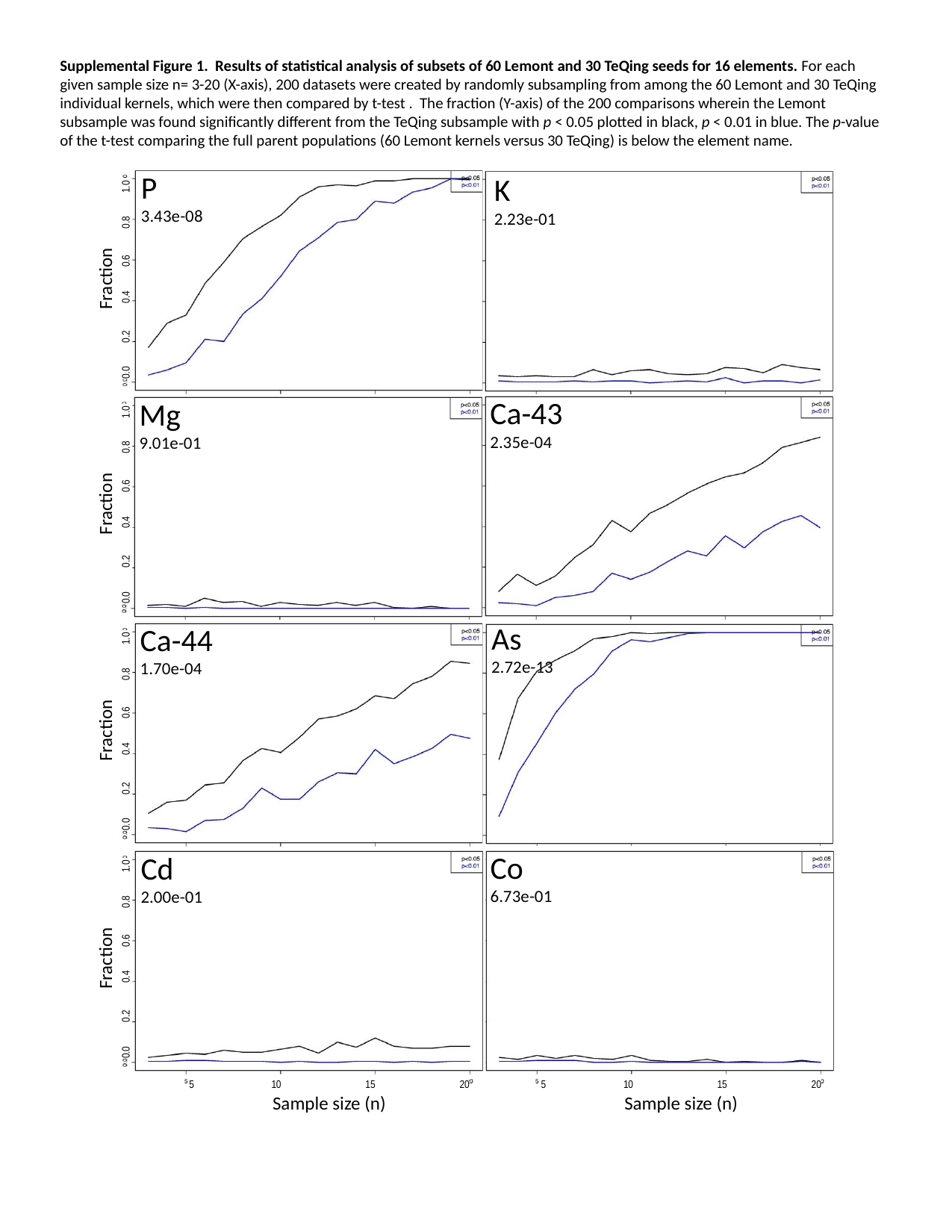

# Supplemental Figure 1. Results of statistical analysis of subsets of 60 Lemont and 30 TeQing seeds for 16 elements. For each given sample size n= 3-20 (X-axis), 200 datasets were created by randomly subsampling from among the 60 Lemont and 30 TeQing individual kernels, which were then compared by t-test . The fraction (Y-axis) of the 200 comparisons wherein the Lemont subsample was found significantly different from the TeQing subsample with p < 0.05 plotted in black, p < 0.01 in blue. The p-value of the t-test comparing the full parent populations (60 Lemont kernels versus 30 TeQing) is below the element name.
P
3.43e-08
K
2.23e-01
Fraction
0.0 0.2 0.4 0.6 0.8 1.0
Ca-43
2.35e-04
Mg
9.01e-01
Fraction
0.0 0.2 0.4 0.6 0.8 1.0
As
2.72e-13
Ca-44
1.70e-04
Fraction
0.0 0.2 0.4 0.6 0.8 1.0
Co
6.73e-01
Cd
2.00e-01
Fraction
0.0 0.2 0.4 0.6 0.8 1.0
5 10 15 20
Sample size (n)
5 10 15 20
Sample size (n)

## Slide 2
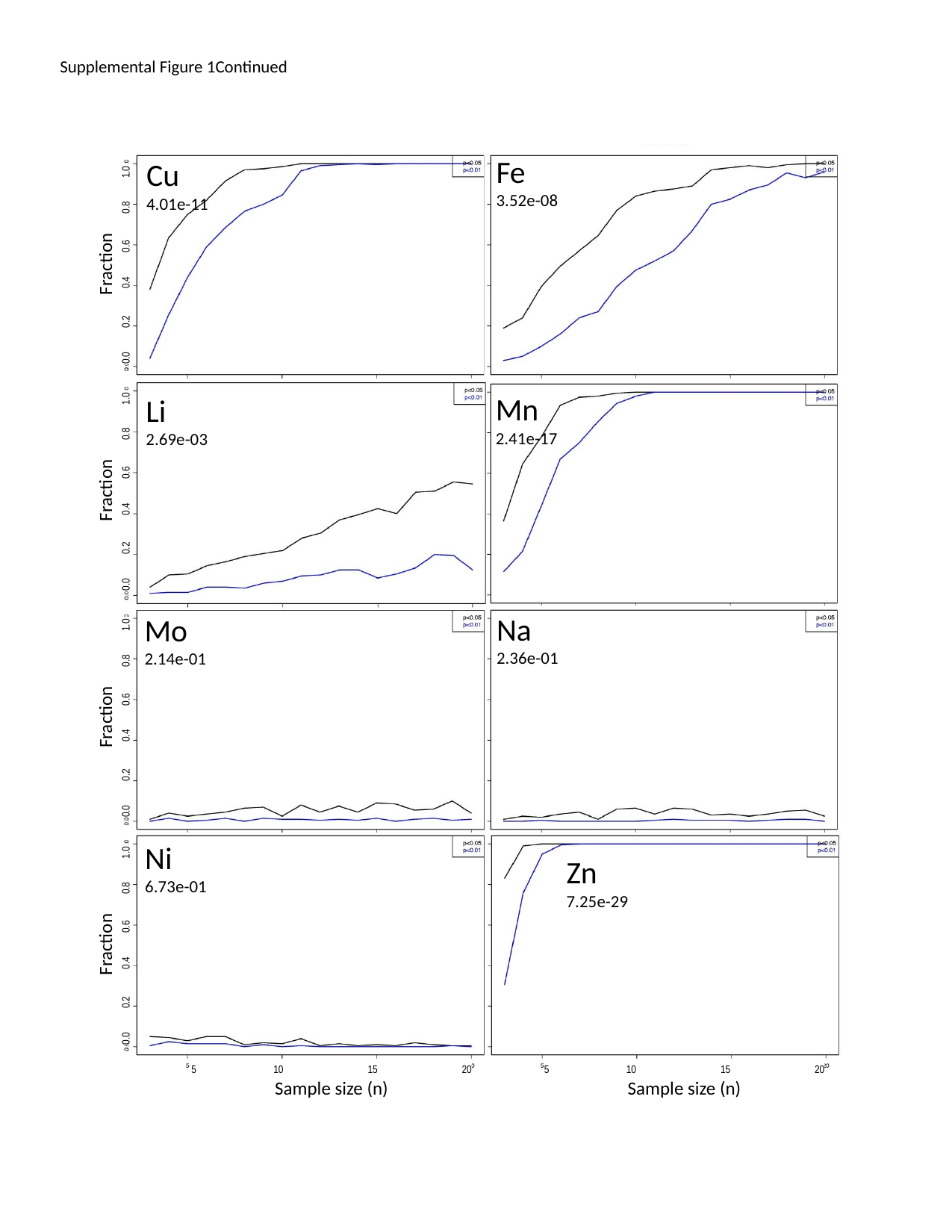

# Supplemental Figure 1Continued
Fe
3.52e-08
Cu
4.01e-11
Fraction
0.0 0.2 0.4 0.6 0.8 1.0
Mn
2.41e-17
Li
2.69e-03
Fraction
0.0 0.2 0.4 0.6 0.8 1.0
Na
2.36e-01
Mo
2.14e-01
Fraction
0.0 0.2 0.4 0.6 0.8 1.0
Ni
6.73e-01
Zn
7.25e-29
Fraction
0.0 0.2 0.4 0.6 0.8 1.0
5 10 15 20
Sample size (n)
5 10 15 20
Sample size (n)
