# Supplementary material for: Mapping and validation of quantitative trait loci associated with concentrations of 16 elements in unmilled rice grain
Source: Theor Appl Genet. 2013 Nov 15;127(1):137–65. doi: 10.1007/s00122-013-2207-5 (PMC4544570; doi:10.1007/s00122-013-2207-5)
Supplement: Supplementary file 2 — Supplementary material 2 (PPT 287 kb) [file 122_2013_2207_MOESM2_ESM.ppt]

## Slide 1
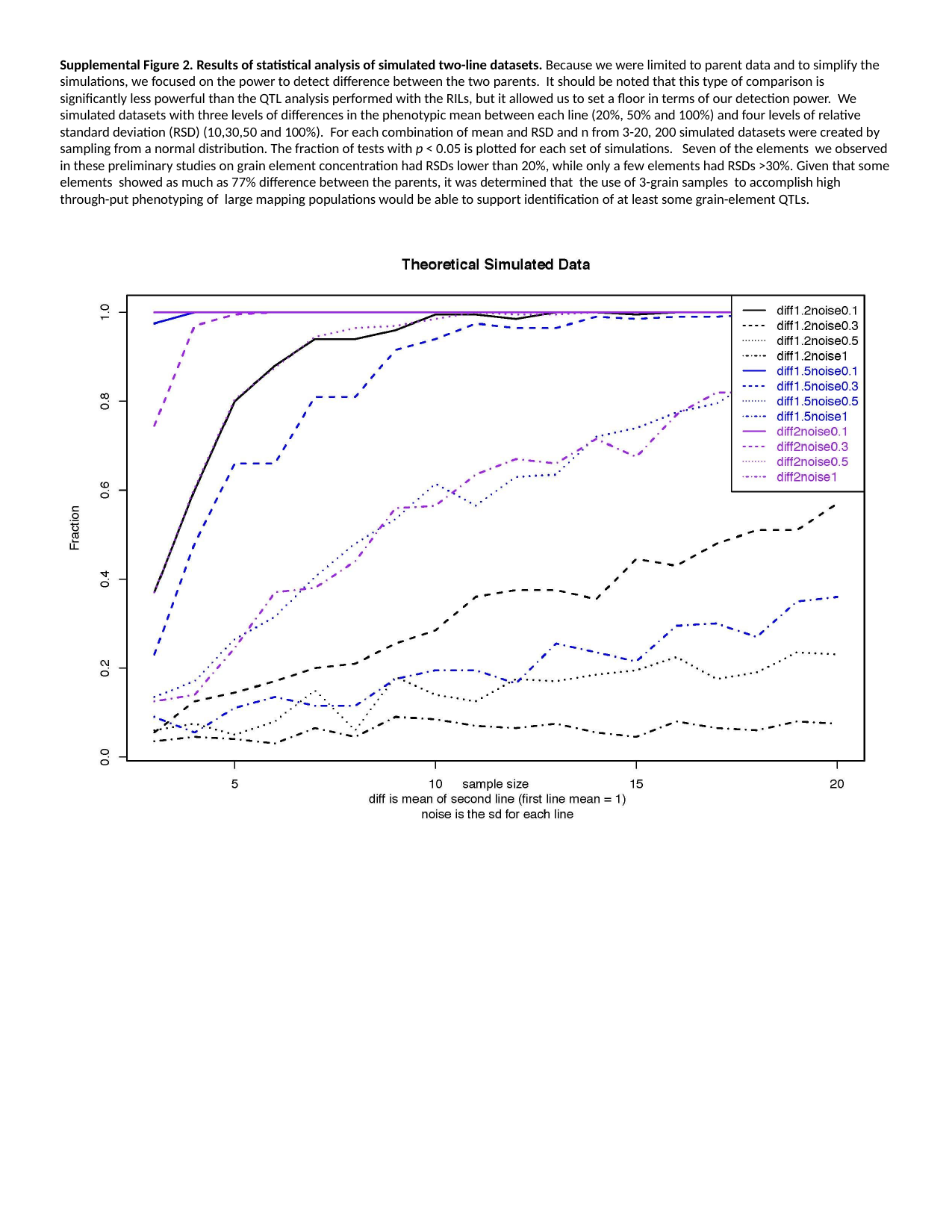

# Supplemental Figure 2. Results of statistical analysis of simulated two-line datasets. Because we were limited to parent data and to simplify the simulations, we focused on the power to detect difference between the two parents. It should be noted that this type of comparison is significantly less powerful than the QTL analysis performed with the RILs, but it allowed us to set a floor in terms of our detection power. We simulated datasets with three levels of differences in the phenotypic mean between each line (20%, 50% and 100%) and four levels of relative standard deviation (RSD) (10,30,50 and 100%). For each combination of mean and RSD and n from 3-20, 200 simulated datasets were created by sampling from a normal distribution. The fraction of tests with p < 0.05 is plotted for each set of simulations. Seven of the elements we observed in these preliminary studies on grain element concentration had RSDs lower than 20%, while only a few elements had RSDs >30%. Given that some elements showed as much as 77% difference between the parents, it was determined that the use of 3-grain samples to accomplish high through-put phenotyping of large mapping populations would be able to support identification of at least some grain-element QTLs.
