# Supplementary material for: Mapping and validation of quantitative trait loci associated with concentrations of 16 elements in unmilled rice grain
Source: Theor Appl Genet. 2013 Nov 15;127(1):137–65. doi: 10.1007/s00122-013-2207-5 (PMC4544570; doi:10.1007/s00122-013-2207-5)
Supplement: Supplementary file 3 — Supplementary material 3 (DOC 278 kb) [file 122_2013_2207_MOESM3_ESM.doc]

***Supplemental Table 1. Trait means ± standard deviations for the grain concentration (ppm) of 16 elements and days to heading observed for the parental lines and the progeny of two mapping populations, LT-RILs and TILs. Continued next page.***

|  |  |  |  |  |  |  |  |  |  |  |  |  |  |  |  |  |
| --- | --- | --- | --- | --- | --- | --- | --- | --- | --- | --- | --- | --- | --- | --- | --- | --- |
|  |  |  | **Macronutrients** | | | | | | | | | | | | |  |
|  |  | in order of concentration (ppm) within plant tissues | | | | | | | | | | | | | | |
|  |  | **P** | | | **K** | | | **Mg** | | | **S** | | | **Ca** | | |
| **Lemont calculated from the seven to 15 repeat plots planted per field replication** | | | | | | | | | | | | | | | | |
| Flooded | 2002 one rep^a^ | -^a^ |  |  | -^a^ |  |  | -^a^ |  |  | -^a^ |  |  | -^a^ |  |  |
|  | 2003 one rep | 4203 | ± | 164 | 2407 | ± | 115 | 1594 | ± | 68 | 953 | ± | 191 | 125 | ± | 11 |
|  | 2006 one rep | 3733 | ± | 148 | 2237 | ± | 116 | 1455 | ± | 63 | 946 | ± | 178 | 117 | ± | 9 |
|  | 2007 rep# 1 | 3878 | ± | 181 | 2251 | ± | 107 | 1505 | ± | 71 | 829 | ± | 26 | 144 | ± | 26 |
|  | 2007 rep# 2 | 4158 | ± | 122 | 2317 | ± | 77 | 1599 | ± | 39 | 1016 | ± | 63 | 127 | ± | 10 |
|  | 2008 rep# 1 | 3652 | ± | 278 | 2800 | ± | 344 | 1485 | ± | 63 | 1086 | ± | 134 | 101 | ± | 14 |
| Unflooded | 2007 rep# 1 | 4314 | ± | 195 | 2469 | ± | 173 | 1667 | ± | 76 | 1312 | ± | 89 | 147 | ± | 12 |
|  | 2008 rep# 1 | 2982 | ± | 315 | 2635 | ± | 334 | 1332 | ± | 98 | 1232 | ± | 268 | 114 | ± | 18 |
|  | 2008 rep# 2 | 3124 | ± | 331 | 2826 | ± | 430 | 1262 | ± | 84 | 1471 | ± | 238 | 125 | ± | 21 |
| **TeQing calculated from the seven to 15 repeat plots planted per field replication** | | | | | | | | | | | | | | | | |
| Flooded | 2002 one rep^a^ | -^a^ |  |  | -^a^ |  |  | -^a^ |  |  | -^a^ |  |  | -^a^ |  |  |
|  | 2003 one rep | 4112 | ± | 194 | 2746 | ± | 176 | 1699 | ± | 86 | 1203 | ± | 196 | 129 | ± | 11 |
|  | 2006 one rep | 3865 | ± | 136 | 2798 | ± | 168 | 1593 | ± | 63 | 1099 | ± | 184 | 126 | ± | 10 |
|  | 2007 rep# 1 | 4110 | ± | 118 | 2623 | ± | 134 | 1735 | ± | 69 | 1115 | ± | 46 | 153 | ± | 31 |
|  | 2007 rep# 2 | 4088 | ± | 187 | 2704 | ± | 103 | 1728 | ± | 57 | 1286 | ± | 66 | 126 | ± | 13 |
|  | 2008 rep# 1 | 3731 | ± | 252 | 3189 | ± | 596 | 1661 | ± | 84 | 1418 | ± | 284 | 114 | ± | 16 |
| Unflooded | 2007 rep# 1 | 3966 | ± | 228 | 2831 | ± | 213 | 1699 | ± | 79 | 1811 | ± | 111 | 130 | ± | 13 |
|  | 2008 rep# 1 | 3051 | ± | 258 | 2933 | ± | 450 | 1386 | ± | 76 | 1397 | ± | 213 | 136 | ± | 35 |
|  | 2008 rep# 2 | 3127 | ± | 332 | 2960 | ± | 413 | 1356 | ± | 75 | 1558 | ± | 238 | 140 | ± | 24 |
| **LT-RILs grown as a single plot per RIL genotype per field replication** | | | | | | | | | | | | | | | | |
| Flooded | 2002 one rep | 4098 | ± | 301 | 2513 | ± | 252 | 1585 | ± | 129 | 1075 | ± | 223 | 127 | ± | 23 |
|  | 2003 one rep | 4053 | ± | 289 | 2449 | ± | 246 | 1576 | ± | 122 | 1098 | ± | 213 | 132 | ± | 26 |
|  | 2006 one rep | 3849 | ± | 307 | 2486 | ± | 278 | 1503 | ± | 131 | 1073 | ± | 225 | 115 | ± | 20 |
|  | 2007 rep# 1^b^ | - ^b^ |  |  | - ^b^ |  |  | - ^b^ |  |  | - ^b^ |  |  | - ^b^ |  |  |
|  | 2007 rep# 2 | 4157 | ± | 295 | 2511 | ± | 250 | 1638 | ± | 126 | 1144 | ± | 129 | 133 | ± | 29 |
|  | 2008 rep# 1 | 3519 | ± | 285 | 3190 | ± | 356 | 1502 | ± | 138 | 1416 | ± | 203 | 102 | ± | 18 |
| **TILs^c^ grown as a single plot per TIL genotype per field replication** | | | | | | | | | | | | | | | | |
| Flooded | 2007 rep# 1 | 4023 | ± | 236 | 2283 | ± | 213 | 1579 | ± | 93 | 920 | ± | 100 | 142 | ± | 35 |
|  | 2007 rep# 2 | 4208 | ± | 283 | 2357 | ± | 208 | 1660 | ± | 119 | 1024 | ± | 99 | 134 | ± | 17 |
|  | 2008 rep# 1 | 3992 | ± | 219 | 2287 | ± | 150 | 1525 | ± | 84 | 945 | ± | 72 | 120 | ± | 14 |
| Unflooded | 2007 rep# 1 | 4396 | ± | 258 | 2563 | ± | 218 | 1713 | ± | 101 | 1385 | ± | 199 | 149 | ± | 18 |
|  | 2008 rep# 1 | 2979 | ± | 383 | 1912 | ± | 195 | 1276 | ± | 122 | 1191 | ± | 156 | 142 | ± | 19 |
|  | 2008 rep# 2 | 3667 | ± | 306 | 2288 | ± | 199 | 1370 | ± | 88 | 1190 | ± | 76 | 155 | ± | 29 |
| a | The 2002, 2003, and 2006 field plots were originally grown for a separate study. No Lemont and TeQing control seed remained from the 2002 harvest which to obtain grain data. Data on days to heading had been collected and are included in the table. | | | | | | | | | | | | | | | |
| b | A majority of the LT-RILs plots planted in the 2007 rep# 1 were too damaged by high winds to obtain sufficient seed of appropriate quality for grain analysis. | | | | | | | | | | | | | | | |
| c | The TIL population was under development and not available for inclusion in this study until 2007. | | | | | | | | | | | | | | | |
| d | This field replication was not evaluated for this particular trait. | | | | | | | | | | | | | | | |

**Supplemental Table 1 continued. Trait means ± standard deviations for the 16 elements and days to heading observed for the parental lines and the progeny of two mapping populations, LT-RILs and TILs. *Continued next page.***

|  |  |  |  |  |  |  |  |  |  |  |  |  |  |  |  |  |  |  |  |
| --- | --- | --- | --- | --- | --- | --- | --- | --- | --- | --- | --- | --- | --- | --- | --- | --- | --- | --- | --- |
|  |  |  | **Micronutrients** | | | | | | | | | | | | | | | |  |
|  |  |  | in alphabetical order | | | | | | | | | | | | | | | |  |
|  |  | **As** | | | **Cd** | | | **Co** | | | **Cu** | | | **Fe** | | | **Mn** | | |
| **Lemont calculated from the seven to 15 repeat plots planted per field replication** | | | | | | | | | | | | | | | | |  |  |  |
| Flooded | 2002 one rep^a^ | -^a^ |  |  | -^a^ |  |  | -^a^ |  |  | -^a^ |  |  | -^a^ |  |  | -^a^ |  |  |
|  | 2003 one rep | 0.205 | ± | 0.070 | 0.035 | ± | 0.025 | 0.035 | ± | 0.010 | 4.38 | ± | 0.33 | 9.42 | ± | 1.41 | 41.9 | ± | 8.0 |
|  | 2006 one rep | 0.538 | ± | 0.148 | 0.011 | ± | 0.002 | 0.027 | ± | 0.005 | 4.23 | ± | 0.24 | 10.40 | ± | 1.01 | 35.4 | ± | 4.7 |
|  | 2007 rep# 1 | 0.561 | ± | 0.116 | 0.008 | ± | 0.003 | 0.043 | ± | 0.010 | 4.32 | ± | 0.44 | 10.51 | ± | 0.91 | 28.0 | ± | 3.9 |
|  | 2007 rep# 2 | 0.409 | ± | 0.187 | 0.009 | ± | 0.003 | 0.045 | ± | 0.006 | 3.28 | ± | 0.39 | 10.25 | ± | 0.66 | 33.3 | ± | 2.8 |
|  | 2008 rep# 1 | 0.545 | ± | 0.134 | 0.015 | ± | 0.004 | 0.026 | ± | 0.008 | 4.13 | ± | 0.25 | 11.23 | ± | 0.67 | 35.4 | ± | 3.5 |
| Unflooded | 2007 rep# 1 | 0.029 | ± | 0.014 | 0.123 | ± | 0.036 | 0.024 | ± | 0.006 | 6.19 | ± | 0.19 | 11.45 | ± | 5.10 | 45.5 | ± | 7.0 |
|  | 2008 rep# 1 | 0.029 | ± | 0.016 | 0.051 | ± | 0.020 | 0.032 | ± | 0.022 | 5.67 | ± | 0.53 | 11.74 | ± | 1.52 | 34.9 | ± | 8.2 |
|  | 2008 rep# 2 | 0.004 | ± | 0.005 | 0.069 | ± | 0.024 | 0.015 | ± | 0.005 | 5.03 | ± | 0.40 | 9.76 | ± | 1.06 | 44.9 | ± | 6.4 |
| **TeQing calculated from the seven to 15 repeat plots planted per field replication** | | | | | | | | | | | | | | | | |  |  |  |
| Flooded | 2002 one rep^a^ | -^a^ |  |  | -^a^ |  |  | -^a^ |  |  | -^a^ |  |  | -^a^ |  |  | -^a^ |  |  |
|  | 2003 one rep | 0.363 | ± | 0.143 | 0.048 | ± | 0.042 | 0.047 | ± | 0.013 | 4.06 | ± | 0.49 | 9.38 | ± | 1.70 | 29.4 | ± | 8.7 |
|  | 2006 one rep | 0.557 | ± | 0.136 | 0.028 | ± | 0.014 | 0.033 | ± | 0.015 | 4.48 | ± | 0.46 | 9.14 | ± | 1.22 | 29.2 | ± | 4.8 |
|  | 2007 rep# 1 | 0.682 | ± | 0.086 | 0.011 | ± | 0.003 | 0.068 | ± | 0.026 | 3.66 | ± | 0.52 | 10.05 | ± | 0.79 | 22.5 | ± | 2.2 |
|  | 2007 rep# 2 | 0.753 | ± | 0.374 | 0.009 | ± | 0.004 | 0.055 | ± | 0.009 | 2.71 | ± | 0.62 | 9.52 | ± | 1.38 | 22.1 | ± | 3.2 |
|  | 2008 rep# 1 | 0.727 | ± | 0.124 | 0.022 | ± | 0.005 | 0.040 | ± | 0.013 | 4.10 | ± | 0.51 | 10.43 | ± | 0.92 | 25.4 | ± | 4.1 |
| Unflooded | 2007 rep# 1 | 0.035 | ± | 0.014 | 0.188 | ± | 0.051 | 0.027 | ± | 0.011 | 6.60 | ± | 0.79 | 11.85 | ± | 6.14 | 25.4 | ± | 4.2 |
|  | 2008 rep# 1 | 0.021 | ± | 0.012 | 0.091 | ± | 0.020 | 0.045 | ± | 0.049 | 5.73 | ± | 0.68 | 10.27 | ± | 1.06 | 28.1 | ± | 8.0 |
|  | 2008 rep# 2 | 0.003 | ± | 0.004 | 0.158 | ± | 0.052 | 0.011 | ± | 0.004 | 6.41 | ± | 0.80 | 8.70 | ± | 1.00 | 34.2 | ± | 5.0 |
| **LT-RILs grown as a single plot per RIL genotype per field replication** | | | | | | | | | | | | | | | | |  |  |  |
| Flooded | 2002 one rep | 0.300 | ± | 0.119 | 0.020 | ± | 0.010 | 0.034 | ± | 0.016 | 3.85 | ± | 0.69 | 10.13 | ± | 1.68 | 28.4 | ± | 6.9 |
|  | 2003 one rep | 0.275 | ± | 0.113 | 0.032 | ± | 0.029 | 0.038 | ± | 0.017 | 3.74 | ± | 0.69 | 10.38 | ± | 1.67 | 32.6 | ± | 8.9 |
|  | 2006 one rep | 0.430 | ± | 0.223 | 0.020 | ± | 0.015 | 0.033 | ± | 0.017 | 4.34 | ± | 1.02 | 10.58 | ± | 1.60 | 30.7 | ± | 7.5 |
|  | 2007 rep# 1^b^ | - ^b^ |  |  | - ^b^ |  |  | - ^b^ |  |  | - ^b^ |  |  | - ^b^ |  |  | - ^b^ |  |  |
|  | 2007 rep# 2 | 0.444 | ± | 0.203 | 0.011 | ± | 0.005 | 0.051 | ± | 0.019 | 3.13 | ± | 0.66 | 9.83 | ± | 4.04 | 28.0 | ± | 6.2 |
|  | 2008 rep# 1 | 0.707 | ± | 0.273 | 0.015 | ± | 0.006 | 0.031 | ± | 0.011 | 3.79 | ± | 0.75 | 11.43 | ± | 1.36 | 27.5 | ± | 5.8 |
| **TILs^c^ grown as a single plot per TIL genotype per field replication** | | | | | | | | | | | | | | | | |  |  |  |
| Flooded | 2007 rep# 1 | 0.665 | ± | 0.180 | 0.006 | ± | 0.003 | 0.050 | ± | 0.016 | 3.89 | ± | 0.57 | 10.51 | ± | 1.23 | 26.9 | ± | 4.6 |
|  | 2007 rep# 2 | 0.680 | ± | 0.216 | 0.007 | ± | 0.003 | 0.043 | ± | 0.012 | 2.68 | ± | 0.52 | 10.27 | ± | 0.98 | 29.0 | ± | 4.3 |
|  | 2008 rep# 1 | 0.642 | ± | 0.195 | 0.013 | ± | 0.004 | 0.030 | ± | 0.009 | 4.18 | ± | 0.52 | 10.55 | ± | 0.85 | 34.2 | ± | 5.8 |
| Unflooded | 2007 rep# 1 | 0.031 | ± | 0.014 | 0.132 | ± | 0.039 | 0.024 | ± | 0.008 | 6.12 | ± | 0.91 | 9.78 | ± | 7.03 | 44.2 | ± | 8.8 |
|  | 2008 rep# 1 | 0.051 | ± | 0.033 | 0.043 | ± | 0.021 | 0.043 | ± | 0.019 | 6.85 | ± | 1.33 | 12.48 | ± | 1.40 | 33.8 | ± | 7.6 |
|  | 2008 rep# 2 | 0.006 | ± | 0.005 | 0.088 | ± | 0.030 | 0.017 | ± | 0.007 | 5.23 | ± | 0.85 | 9.32 | ± | 1.15 | 45.5 | ± | 10.6 |
| a | The 2002, 2003, and 2006 field plots were originally grown for a separate study. No Lemont and TeQing control seed remained from the 2002 harvest which to obtain grain data. Data on days to heading had been collected and are included in the table. | | | | | | | | | | | | | | | | | | |
| b | A majority of the LT-RILs plots planted in the 2007 rep# 1 were too damaged by high winds to obtain sufficient seed of appropriate quality for grain analysis. | | | | | | | | | | | | | | | | | | |
| c | The TIL population was under development and not available for inclusion in this study until 2007. | | | | | | | | | | | | | | | | | | |
| d | This field replication was not evaluated for this particular trait. | | | | | | | | | | | | | | | | | | |

**Supplemental Table 1 continued. Trait means ± standard deviations for the 16 elements and days to heading observed for the parental lines and the progeny of two mapping populations, LT-RILs and TILs.**

|  |  |  |  |  |  |  |  |  |  |  |  |  |  |  |  |  |  |  |  |
| --- | --- | --- | --- | --- | --- | --- | --- | --- | --- | --- | --- | --- | --- | --- | --- | --- | --- | --- | --- |
|  |  | **Micronutrients continued** | | | | | | | | | | | | | |  | **Days to** | | |
|  |  | in alphabetical order | | | | | | | | | | | | | |  | **Heading** | | |
|  |  | **Mo** | | | **Ni** | | | **Rb** | | | **Sr** | | | **Zn** | | |  |  |  |
| **Lemont calculated from the seven to 15 repeat plots planted per field replication** | | | | | | | | | | | | | | | | |  |  |  |
| Flooded | 2002 one rep^a^ | -^a^ |  |  | -^a^ |  |  | -^a^ |  |  | -^a^ |  |  | -^a^ |  |  | 85.8 | ± | 8.6 |
|  | 2003 one rep | 0.396 | ± | 0.051 | 0.058 | ± | 0.016 | 6.6 | ± | 1.5 | 0.614 | ± | 0.087 | 28.9 | ± | 1.6 | 84.4 | ± | 7.4 |
|  | 2006 one rep | 0.555 | ± | 0.047 | 0.060 | ± | 0.012 | 11.3 | ± | 3.1 | 0.565 | ± | 0.079 | 27.4 | ± | 1.1 | 95.4 | ± | 6.9 |
|  | 2007 rep# 1 | 0.452 | ± | 0.051 | 0.047 | ± | 0.007 | 12.6 | ± | 1.5 | 0.627 | ± | 0.105 | 29.1 | ± | 1.7 | 99.4 | ± | 4.6 |
|  | 2007 rep# 2 | 0.484 | ± | 0.059 | 0.034 | ± | 0.007 | 7.7 | ± | 1.5 | 0.536 | ± | 0.062 | 28.0 | ± | 1.7 | - ^d^ |  |  |
|  | 2008 rep# 1 | 0.485 | ± | 0.035 | 1.133 | ± | 0.708 | 10.1 | ± | 2.1 | 0.533 | ± | 0.041 | 24.5 | ± | 2.1 | 90.6 | ± | 4.8 |
| Unflooded | 2007 rep# 1 | 0.521 | ± | 0.055 | 0.332 | ± | 0.031 | 10.8 | ± | 2.7 | - ^d^ |  |  | 33.9 | ± | 6.9 | 96.3 | ± | 3.6 |
|  | 2008 rep# 1 | 0.380 | ± | 0.060 | 10.940 | ± | 4.786 | 10.2 | ± | 2.0 | 0.443 | ± | 0.089 | 25.5 | ± | 1.7 | 89.9 | ± | 2.6 |
|  | 2008 rep# 2 | 0.369 | ± | 0.047 | 9.725 | ± | 6.144 | 7.8 | ± | 1.5 | 0.369 | ± | 0.032 | 23.4 | ± | 2.0 | - ^d^ |  |  |
| **TeQing calculated from the seven to 15 repeat plots planted per field replication** | | | | | | | | | | | | | | | | |  |  |  |
| Flooded | 2002 one rep^a^ | -^a^ |  |  | - ^a^ |  |  | - ^a^ |  |  | - ^a^ |  |  | - ^a^ |  |  | 87.0 | ± | 9.6 |
|  | 2003 one rep | 0.362 | ± | 0.040 | 0.058 | ± | 0.024 | 12.6 | ± | 3.7 | 0.669 | ± | 0.155 | 22.2 | ± | 1.2 | 90.5 | ± | 8.4 |
|  | 2006 one rep | 0.415 | ± | 0.052 | 0.068 | ± | 0.016 | 18.3 | ± | 4.2 | 0.600 | ± | 0.072 | 22.5 | ± | 2.4 | 97.3 | ± | 7.9 |
|  | 2007 rep# 1 | 0.387 | ± | 0.046 | 0.040 | ± | 0.013 | 14.1 | ± | 2.0 | 0.617 | ± | 0.135 | 22.4 | ± | 2.3 | 100.2 | ± | 4.3 |
|  | 2007 rep# 2 | 0.486 | ± | 0.067 | 0.029 | ± | 0.010 | 13.2 | ± | 2.5 | 0.494 | ± | 0.040 | 20.9 | ± | 1.6 | - ^d^ |  |  |
|  | 2008 rep# 1 | 0.393 | ± | 0.050 | 1.004 | ± | 0.823 | 12.0 | ± | 1.9 | 0.579 | ± | 0.086 | 20.6 | ± | 1.7 | 92.7 | ± | 4.2 |
| Unflooded | 2007 rep# 1 | 0.458 | ± | 0.057 | 0.381 | ± | 0.232 | 17.8 | ± | 5.8 | - ^d^ |  |  | 28.4 | ± | 2.0 | 103.5 | ± | 1.3 |
|  | 2008 rep# 1 | 0.285 | ± | 0.056 | 8.238 | ± | 6.690 | 14.3 | ± | 3.0 | 0.636 | ± | 0.129 | 24.1 | ± | 4.5 | 94.7 | ± | 1.8 |
|  | 2008 rep# 2 | 0.298 | ± | 0.030 | 8.733 | ± | 7.734 | 7.4 | ± | 1.1 | 0.347 | ± | 0.036 | 24.0 | ± | 3.1 | - ^d^ |  |  |
| **LT-RILs grown as a single plot per RIL genotype per field replication** | | | | | | | | | | | | | | | | |  |  |  |
| Flooded | 2002 one rep | 0.414 | ± | 0.081 | 0.058 | ± | 0.019 | 8.9 | ± | 3.1 | 0.564 | ± | 0.166 | 26.7 | ± | 3.4 | 88.5 | ± | 12.4 |
|  | 2003 one rep | 0.415 | ± | 0.089 | 0.060 | ± | 0.032 | 8.0 | ± | 2.3 | 0.693 | ± | 0.205 | 26.6 | ± | 3.2 | 91.9 | ± | 9.7 |
|  | 2006 one rep | 0.460 | ± | 0.108 | 0.080 | ± | 0.041 | 13.0 | ± | 5.5 | 0.546 | ± | 0.154 | 27.6 | ± | 3.7 | 102.6 | ± | 12.3 |
|  | 2007 rep# 1^b^ | - ^b^ |  |  | - ^b^ |  |  | - ^b^ |  |  | - ^b^ |  |  | - ^b^ |  |  | - ^b^ |  |  |
|  | 2007 rep# 2 | 0.500 | ± | 0.096 | 0.037 | ± | 0.015 | 10.1 | ± | 2.5 | 0.534 | ± | 0.128 | 25.0 | ± | 3.3 | - ^d^ |  |  |
|  | 2008 rep# 1 | 0.427 | ± | 0.089 | 1.837 | ± | 0.534 | 11.0 | ± | 2.7 | 0.543 | ± | 0.133 | 21.9 | ± | 2.9 | 93.0 | ± | 9.4 |
| **TILs^c^ grown as a single plot per TIL genotype per field replication** | | | | | | | | | | | | | | | | |  |  |  |
| Flooded | 2007 rep# 1 | 0.443 | ± | 0.069 | 0.051 | ± | 0.017 | 11.8 | ± | 2.6 | 0.629 | ± | 0.159 | 29.3 | ± | 4.1 | 99.4 | ± | 8.4 |
|  | 2007 rep# 2 | 0.547 | ± | 0.115 | 0.031 | ± | 0.010 | 9.2 | ± | 1.5 | 0.598 | ± | 0.116 | 27.1 | ± | 2.5 | - ^d^ |  |  |
|  | 2008 rep# 1 | 0.489 | ± | 0.077 | 0.036 | ± | 0.010 | 11.6 | ± | 2.3 | 0.574 | ± | 0.131 | 27.8 | ± | 3.0 | 90.8 | ± | 6.6 |
| Unflooded | 2007 rep# 1 | 0.474 | ± | 0.072 | 0.343 | ± | 0.056 | 10.6 | ± | 3.1 | - ^d^ |  |  | 34.1 | ± | 3.9 | 98.6 | ± | 7.2 |
|  | 2008 rep# 1 | 0.357 | ± | 0.081 | 0.303 | ± | 0.051 | 10.0 | ± | 2.6 | 0.481 | ± | 0.083 | 34.5 | ± | 5.1 | 93.7 | ± | 6.2 |
|  | 2008 rep# 2 | 0.359 | ± | 0.075 | 0.329 | ± | 0.053 | 7.9 | ± | 1.6 | 0.396 | ± | 0.069 | 27.7 | ± | 3.0 | - ^d^ |  |  |
| a | The 2002, 2003, and 2006 field plots were originally grown for a separate study. No Lemont and TeQing control seed remained from the 2002 harvest which to obtain grain data. Data on days to heading had been collected and are included in the table. | | | | | | | | | | | | | | | | | | |
| b | A majority of the LT-RILs plots planted in the 2007 rep# 1 were too damaged by high winds to obtain sufficient seed of appropriate quality for grain analysis. | | | | | | | | | | | | | | | | | | |
| c | The TIL population was under development and not available for inclusion in this study until 2007. | | | | | | | | | | | | | | | | | | |
| d | This field replication was not evaluated for this particular trait. | | | | | | | | | | | | | | | | | | |
