# Supplementary material for: Mapping and validation of quantitative trait loci associated with concentrations of 16 elements in unmilled rice grain
Source: Theor Appl Genet. 2013 Nov 15;127(1):137–65. doi: 10.1007/s00122-013-2207-5 (PMC4544570; doi:10.1007/s00122-013-2207-5)
Supplement: Supplementary file 4 — Supplementary material 4 (DOC 67 kb) [file 122_2013_2207_MOESM4_ESM.doc]

**Supplemental Table 2.** Pairwise correlations between years for the 16 elements among the LT-RILs. The time-staggered planting of LT-RILs according to their maturity in 2002, 2003, and 2006 shortened the range in flowering time across the population to calendar three weeks, as opposed to 12 weeks between the earliest and latest maturing LT-RILs when planted on the same day, as in 2007 and 2008.

|  |  |  |  |  |  |  |  |  |  |  |  |  |  |
| --- | --- | --- | --- | --- | --- | --- | --- | --- | --- | --- | --- | --- | --- |
|  | **Correlations between years with similar planting method** | | | |  | **Correlations between years where different planting methods were used per year** | | | | | |  | **Avg. across years** |
|  | **2002 × 2003** | **2002 × 2006** | **2003 × 2006** | **2007 × 2008** |  | **2002 × 2007** | **2003 × 2007** | **2006 × 2007** | **2002 × 2008** | **2003 × 2008** | **2006 × 2008** |  |
| **Avg. across elements** | 0.43 | 0.36 | 0.34 | 0.26 |  | 0.31 | 0.25 | 0.30 | 0.27 | 0.25 | 0.23 |  | 0.30 |
| **P** | 0.51 | 0.26 | 0.38 | 0.23 |  | 0.43 | 0.40 | 0.42 | 0.02 | 0.13 | 0.28 |  | 0.31 |
| **K** | 0.57 | 0.47 | 0.61 | 0.48 |  | 0.59 | 0.53 | 0.62 | 0.17 | 0.25 | 0.42 |  | 0.47 |
| **Mg** | 0.46 | 0.41 | 0.51 | 0.37 |  | 0.51 | 0.53 | 0.49 | 0.29 | 0.28 | 0.35 |  | 0.42 |
| **S** | 0.12 | 0.04 | 0.15 | 0.34 |  | 0.37 | 0.25 | 0.34 | 0.24 | 0.19 | 0.09 |  | 0.21 |
| **Ca** | 0.56 | 0.48 | 0.41 | 0.20 |  | 0.31 | 0.28 | 0.44 | 0.35 | 0.30 | 0.19 |  | 0.35 |
| **As** | 0.34 | 0.32 | 0.24 | 0.16 |  | 0.03 | 0.05 | 0.04 | 0.25 | 0.24 | 0.01 |  | 0.17 |
| **Cd** | 0.38 | 0.21 | 0.15 | 0.14 |  | 0.13 | 0.13 | 0.02 | 0.22 | 0.25 | 0.08 |  | 0.17 |
| **Co** | 0.58 | 0.61 | 0.51 | 0.09 |  | 0.41 | 0.45 | 0.41 | 0.08 | 0.25 | 0.15 |  | 0.35 |
| **Cu** | 0.63 | 0.32 | 0.57 | 0.24 |  | 0.33 | 0.26 | 0.25 | 0.34 | 0.29 | 0.34 |  | 0.36 |
| **Fe** | 0.29 | 0.15 | 0.35 | 0.01 |  | 0.18 | 0.13 | 0.01 | 0.14 | 0.22 | 0.37 |  | 0.19 |
| **Mn** | 0.51 | 0.32 | 0.18 | 0.37 |  | 0.18 | 0.14 | 0.46 | 0.15 | 0.09 | 0.20 |  | 0.26 |
| **Mo** | 0.61 | 0.54 | 0.55 | 0.43 |  | 0.43 | 0.40 | 0.41 | 0.28 | 0.32 | 0.40 |  | 0.44 |
| **Ni** | 0.22 | 0.21 | 0.05 | 0.24 |  | 0.33 | 0.03 | 0.06 | 0.26 | 0.03 | 0.09 |  | 0.15 |
| **Rb** | 0.21 | 0.57 | 0.20 | 0.49 |  | 0.35 | 0.29 | 0.39 | 0.38 | 0.29 | 0.41 |  | 0.36 |
| **Sr** | 0.72 | 0.55 | 0.53 | 0.30 |  | 0.43 | 0.30 | 0.58 | 0.53 | 0.44 | 0.33 |  | 0.47 |
| **Zn** | 0.49 | 0.36 | 0.49 | 0.44 |  | 0.35 | 0.44 | 0.34 | 0.28 | 0.29 | 0.21 |  | 0.37 |
|  |  |  |  |  |  |  |  |  |  |  |  |  |  |
